# Development of a core outcome set for amblyopia, strabismus and ocular motility disorders: a review to identify outcome measures

**DOI:** 10.1186/s12886-019-1055-8

**Published:** 2019-02-08

**Authors:** Samia Al Jabri, Jamie Kirkham, Fiona J. Rowe

**Affiliations:** 1 Department of Health Services Research, University of Liverpool, Waterhouse Building Block B, 2nd Floor, 1-3 Brownlow Street, L69 3GL Liverpool, UK; 2 Department of Biostatistics, University of Liverpool, Liverpool, UK

**Keywords:** Amblyopia, Strabismus, Ocular motility disorders, Outcome measures, Core outcome set

## Abstract

**Background:**

Core Outcome Sets (COS) are defined as the minimum sets of outcomes that should be measured and reported in all randomised controlled trials to facilitate combination and comparability of research. The aim of this review is to produce an item bank of previously reported outcome measures from published studies in amblyopia, strabismus and ocular motility disorders to initiate the development of COS.

**Methods:**

A review was conducted to identify articles reporting outcome measures for amblyopia, strabismus and ocular motility disorders. Using systematic methods according to the COMET handbook we searched key electronic bibliographic databases from 1st January 2011 to 27th September 2016 using MESH terms and alternatives indicating the different subtypes of amblyopia, strabismus and ocular motility disorders in relation to treatment outcomes and all synonyms. We included Cochrane reviews, other systematic reviews, controlled trials, non-systematic reviews and retrospective studies. Data was extracted to tabulate demographics of included studies, primary and secondary outcomes, methods of measurement and their time points.

**Results:**

A total of 142 studies were included; 42 in amblyopia, 33 in strabismus, and 68 in ocular motility disorders (one study overlap between amblyopia and strabismus). We identified ten main outcome measure domains for amblyopia, 14 for strabismus, and ten common “visual or motility” outcome measure domains for ocular motility disorders. Within the domains, we found variable nomenclature being used and diversity in methods and timings of measurements.

**Conclusion:**

This review highlights discrepancies in outcome measure reporting within published literature for amblyopia, strabismus and ocular motility and it generated an item bank of the most commonly used and reported outcome measures for each of the three conditions from recent literature to start the process of COS development. Consensus among all stakeholders including patients and professionals is recommended to establish a useful COS.

**Electronic supplementary material:**

The online version of this article (10.1186/s12886-019-1055-8) contains supplementary material, which is available to authorized users.

## Background

Amblyopia, strabismus and ocular motility disorders occur in about 10% of the general population (amblyopia 2–5%, strabismus 4%) [1]. They often present as childhood conditions and can constitute long-term problems for children and young adults. Strabismus and ocular motility disorders can also develop as acquired conditions due to neurological, endocrine and traumatic causes. There are several approaches to the management of these conditions including occlusion, penalisation, spectacles, prisms, drugs, surgery, botulinum toxin, exercises, watchful waiting, or a combination of two or more of the above [2]. The effects from these treatments such as improvements in symptoms or side effects are assessed by outcome measures and are usually used to formally evaluate management options in clinical studies. However varied outcome measures and several endpoints are often used [3–5]. This lack of standardisation makes it difficult to compare the conclusions of these studies and, as a result, renders it challenging to discuss realistically the likely outcomes of treatment with patients in the clinic [6].

One strategy suggested to overcome the issues resulting from variable outcome measures is the development of Core Outcome Sets (COS). This is defined as the minimum set of outcomes that should be measured and reported in all randomised controlled trials [7]. The COS will make it easier for the results of trials to be compared, contrasted and combined, lead to research that is more likely to have measured relevant outcomes due to involvement of relevant stakeholders, and enhance the value of evidence synthesis by ensuring that all trials contribute usable information [7]. Therefore, it is postulated that the use of COS would increase the potential in carrying out future meta-analysis for target conditions.

The numerous and diverse outcome measures that may be used for amblyopia, strabismus and ocular motility disorders include, amongst others, visual acuity, angle of deviation, range of ocular movements, fixation stability and binocular vision measurements. There are a number of Cochrane systematic reviews that consider a range of treatment trials for amblyopia, strabismus and ocular motility disorders. Their recommendations call for clarification of dose/response effect and further investigation of treatment regimens [2–4]. An attempt to utilise a COS is evident for the National Strabismus Data Set project [8]. A recent review recommended four outcomes for reporting results of surgery for intermittent exotropia [5] but was limited by the extent of literature review and lack of external consensus. A short narrative review of outcome measurements for size of deviation showed considerable variability across the tests available and the recommendations for their use [9].

Development of a COS involves a number of stages that commence with a systematic review of the literature to identify existing knowledge about outcome measures [7]. This is then followed by qualitative studies, Delphi surveys to consult widely on outcome measures and finally, consensus meetings to discuss and agree on the COS [7]. This paper reports the first stage – the literature review to identify the reported range of outcome measures in the published literature for amblyopia, strabismus and ocular motility disorders.

### Objectives

The primary aim of this review is to generate an item bank of relevant outcome measures previously reported by researchers and clinicians in studies of treatment of conditions under evaluation. The review aims also to determine the variation in measuring methods used and timings of assessments.

The secondary objectives of this review are to investigate sources of variability of outcome measure definitions including different age groups, study designs, types of amblyopia (e.g. refractive, strabismic, stimulus deprivation), types of strabismus (e.g. exotropia, esotropia), and types of ocular motility disorder (e.g. accommodation and convergence disorders, mechanical restrictions, myogenic, neurogenic, nystagmus, patterns deviation and gaze palsy).

## Methods

A protocol for the development of this COS project was written by a steering committee – a team of stakeholders including COS developers, ophthalmologists, orthoptists and journal editors. The review protocol was registered in the COMET initiative website (http://www.comet-initiative.org/studies/details/900?result=true) and published as open access (http://pcwww.liv.ac.uk/~rowef/index_files/Page356.htm). The review, using systematic rigorous methods, was conducted in accordance with the guidelines from the COMET handbook [7]. A PRISMA checklist [10] has been completed for the systematic review and can be found in Additional file 1 : Table S1.

### Eligibility criteria

#### Age

Subjects of all ages with target conditions were included.

Target conditions:

1. Amblyopia (unilateral, bilateral) of any type or severity (refractive, meridional, ametropic, strabismic or stimulus deprivation).
2. Strabismus (latent, manifest, constant, intermittent, micro) of any type and severity (eso, exo, hyper, hypo, cyclo deviation)
3. Ocular motility disorders (OMDs) of any type and severity (nystagmus, horizontal/vertical gaze palsy, cranial nerve palsy, convergence/divergence disorder, patterns of horizontal incomitance, mechanical restrictions, myogenic disorders like thyroid eye disease and myasthenia with ocular involvement).

We included all three target conditions in recognition of the considerable overlap between them, for example amblyopia and strabismus often coexist with presentation in childhood with frequent persistence to adult life; whilst strabismus and ocular motility disorders often coexist with onset at any age through childhood and adult life.

#### Interventions

We included any intervention that aimed to improve the conditions of amblyopia, strabismus and ocular motility disorders or alleviate their associated visual symptoms. Interventions may include prisms, occlusion, optical penalisation, glasses, exercises, behavioural vision training, extraocular muscle surgery, extraocular muscle injection of botulinum toxin, pharmacology therapy, and watchful waiting/observation.

#### Comparisons

We included any comparison between the effectiveness of a treatment modality with another or with no treatment for each condition.

#### Outcome measures

We included any reported outcome measure that was recorded using any possible instrument or method at any point of time from the intervention.

#### Types of studies

The following types of studies were considered to be included in this review:

• Cochrane systematic reviews

• Systematic reviews (with or without meta-analysis) inclusive of diagnostic test accuracy reviews

• Randomised controlled trials (RCT)

• Controlled clinical trials (CCT)

• Cohort studies

• Case series with > 10 subjects

We excluded all case reports and letters/editorials.

### Search methods for identification of studies

We used systematic strategies to search key electronic databases. We searched Cochrane registers and electronic bibliographic databases including CENTRAL, ovid MEDLINE, SCOPUS, CINAHL, AMED and PsycINFO with search dates of 1st January 2011 through to 27th of September 2016. This period was selected given the considerable increase in studies, trials and reviews in recent years and to extract treatment outcome measures that are relevant to recent research and clinical practice. As per COMET handbook guidance [7] we recognised that overly large reviews would be resource intensive and might not yield important additional outcomes.

We did not search for unpublished studies or in clinical trials registries and we did not hand-search any additional resources. We performed citation tracking using Web of Science Cited Reference Search for all included studies and searched the reference lists of included trials and review articles. Studies identified from the combined search were exported to an EndNoteX7 library. Search terms included a comprehensive range of MeSH terms and alternatives.

SJ and senior author FR developed the table of search terms jointly to include all target conditions and all synonyms of outcome measures, outcomes or assessments. Appropriate Boolean operators were obtained using University of Liverpool library online resources. Whenever available, the filters of “limit to humans” and “exclude case reports” were applied to the search in the databases. An example for search terms for one database is outlined in Additional file 2: Table S2. There was no language restriction while carrying out the search. The search strategy was discussed with and approved by the study steering committee.

### Selection of studies

During the first stage of selection, SJ screened the titles and abstracts identified from the search that had been exported to an EndNoteX7 database. Senior researchers (FR and JJK) were consulted when there was a doubt about any abstract. Full text papers were accessed for all papers whose title and/or abstract met the eligibility criteria. These full text papers of potentially relevant studies were considered in the second stage of selection in which the selection criteria were again applied to the full paper content. We resolved disagreements by discussion.

**Fig. 1:**
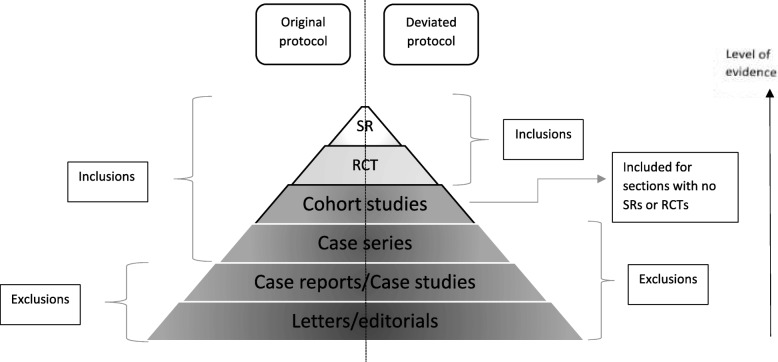
Flowchart of selection process

The study protocol was registered in the COMET initiative website. We planned to include systematic reviews, controlled trials, non-systematic reviews, prospective and retrospective cohort studies as well as case series with > 10 subjects at the time of writing the protocol for this systematic review. However this was not done in the actual review (protocol deviation) due to the excessive number of studies that met the inclusion criteria from the higher quality papers of systematic reviews and RCTs/CCTs for most conditions (Fig. 1).

Only a sample of non-systematic reviews and cohort studies was used (as the next best evidence quality to RCTs/SRs) to supplement this review when the number of studies from RCT/SRs for a particular sub-condition was sparse. We performed this also to check for any potentially important missed outcome measures from RCTs/SRs, e.g. long-term outcome measures or adverse events. The sample was variable depending on the availability of articles within the search results pertaining to a certain condition. The sample was increased until outcome measure saturation was achieved, defined as when no additional new measures could be identified and they were repetitive across studies. One non-systematic review and four retrospective studies for the ocular motility disorder sub-condition “pattern deviation” were included as we could not identify any relevant RCTs/SRs from the search results.

### Data extraction

SJ extracted the data using a pre-determined data extraction form. Senior reviewer FR reviewed 20% of studies to confirm fulfilling data extraction. There were no disagreements or inconsistencies.

The following data was extracted from each study:

- Demographics
  1. Study type.
  2. Author details.
  3. Year and journal of publication.
  4. Country where study was conducted.
  5. Condition(s) under investigation (amblyopia/strabismus/ocular motility disorder).
  6. Age of participants in the study population.
- Outcome measures
  1. The designated outcome measure (primary and secondary).
  2. Outcome measurements (methods or instruments of measurements).
  3. The time points at which they were measured.

### Data analysis and presentation

All data was extracted verbatim from the source manuscripts to facilitate external critical review of the COS right back to its inception. Different nomenclature or aspects used to indicate the same outcome measure were grouped within main outcome headings (domains) when applicable to facilitate easy classification of outcome measures. For example for amblyopia the following aspects were recorded under the outcome measure heading of visual acuity (VA): best corrected visual acuity (BCVA), near visual acuity and binocular visual acuity. They were all recorded as reported in individual studies and then grouped together under one main outcome measure (VA). The method of measurement for BCVA was reported; e.g. using “Electronic Early Treatment Diabetic Retinopathy Study (ETDRS) VA protocol” or “Snellen chart” etc. and in addition we recorded the time when the measurement was made.

A similar classification and tabulation of information regarding the different outcome measures for the different conditions and sub-conditions was used. For the purpose of this study we did not perform a quality assessment for outcome data from the included studies as we sought only to create an item bank of all utilised outcome measures and outcome measurements. Hence a critique of the methodological quality of the studies was not necessary [7].

We generated an item bank of relevant outcome measures for amblyopia, strabismus and ocular motility disorders presented in percentages of frequency in included studies. In addition we produced an inventory of methods of measurements and their timings. Ocular motility disorders outcome measures were further stratified by sub-condition.

## Results

### Study selection

**Fig. 2:**
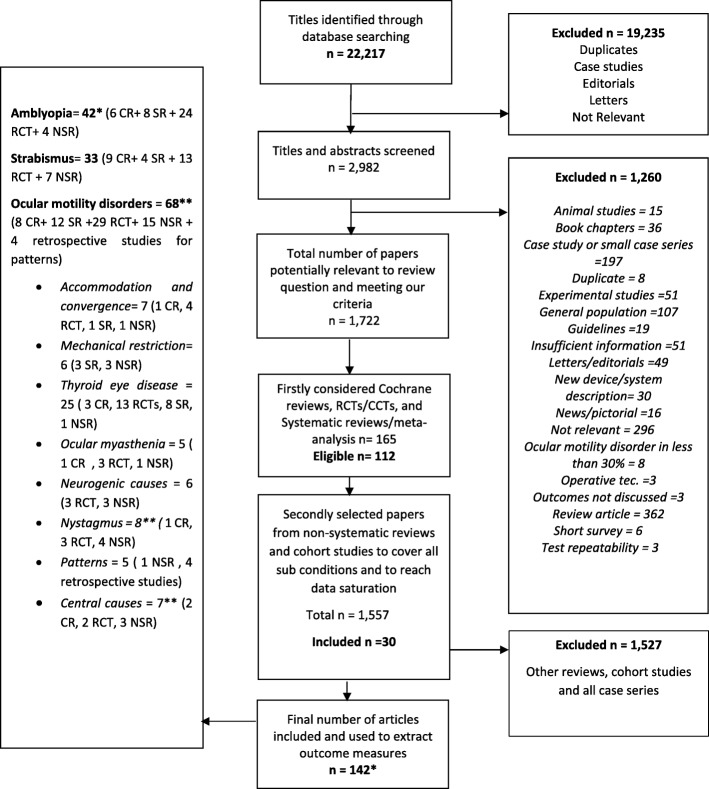
Study search

Electronic search of the six databases returned 22,217 hits, which were exported to the reference manager “EndNote X7”. Titles were screened and the number reduced after removing duplicates and non-relevant papers to 2982 reports (Fig. 2). Another 1260 papers were excluded after screening the abstracts.

We were left with 1722 potentially relevant reports to our review question and meeting our eligibility criteria in review protocol (systematic reviews, controlled trials, cohort studies, and case series with > 10 patients for target conditions and populations). Due to the large number of the potently eligible papers, we considered a modification to our eligibility criteria stated previously in the study protocol. We consulted the COMET handbook in which it is suggested, as an option, to perform the systematic review in stages to check if outcome saturation is reached [7] We took a decision, as a first stage analysis (protocol deviation, Fig. 1), to include only systematic reviews and controlled trials initially. This presented us with a total of 165 studies. Out of those, 53 studies were excluded after reading full articles due to irrelevance or lack of “visual or ocular motility” outcomes leaving us with 112 eligible systematic reviews and trials.

Then, when no systematic reviews or trials were found to cover a particular sub condition, cohort studies were considered as the next stage of the analysis. Moreover, we included additional non-systematic reviews distributed across the different conditions and sub conditions of motility disorders to ensure a comprehensive literature review and data saturation. The included number of both cohort studies and non-systematic reviews was 30 in total (4 cohort and 26 non-systematic reviews).

**Fig. 3:**
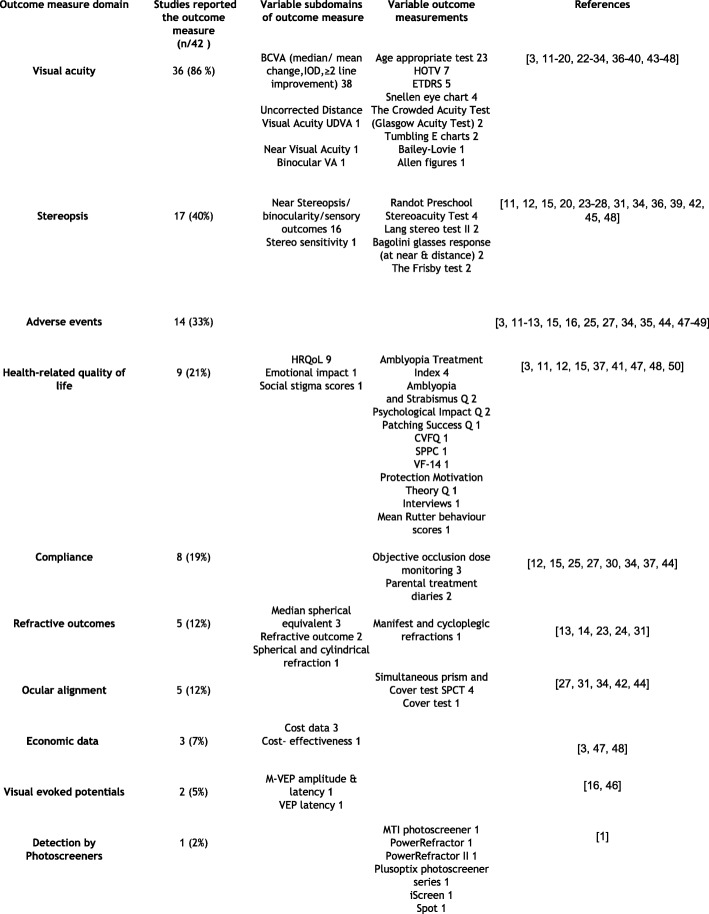
Distribution of included studies by countries where they were conducted

The total number of studies included in analysis in this review eventually was 142 unique studies. The studies came from a wide range of countries with predominance from the United States, the United Kingdom, China and various European countries (Fig. 3).

The following sections will present our findings individually for each of the three conditions: Amblyopia, Strabismus and OMDs outlining types of included studies, types of the conditions, age groups and treatments and listing outcome measures, measurements and commenting on timings. Further subgroup analysis is carried out for OMDs sub-conditions.

### Amblyopia

#### Types of included studies

In this review we looked at a total of 42 studies in amblyopia including six Cochrane reviews, eight systematic reviews and meta-analysis, 24 controlled trials and four non-systematic reviews.

#### Types of amblyopia and included age groups

The types of amblyopia targeted in included studies ranged from childhood amblyopia [1, 11–42] to residual amblyopia in older children [43, 44], adolescents or adults [40, 41, 45, 46], unilateral [3, 12] and bilateral [3], refractive [3], anisometropic [19, 23], strabismic [24, 47], and stimulus deprivation amblyopia [48].

#### Types of treatment

Interventions varied from the “gold standard” refractive correction and occlusion or atropine penalization [24, 27, 30, 31] to the more modern controversial treatments such as low-level laser [46], photic stimulation [43], and medical and behavioural treatment [45] which were more likely to be used beyond the visual maturation age when conventional treatments often fail.

Occlusion dosages and approaches were investigated in a number of included studies such as part-time versus full time occlusion [38], personalized versus standardized [33], and occlusion versus Bangerter filters [23]. Atropine penalization versus patching, and atropine combined with plano lenses were investigated in three of the included studies [20, 31, 34].

Binocular training with interactive computerized games or video clips versus monocular occlusion treatment were under investigation in seven studies [12, 22, 25, 28, 35, 36, 40]. Levodopa was the main treatment used in two studies [16, 44] and Citocolin combined with patching was used to treat residual amblyopia in older children in one of the included studies [29]. Acupuncture and Chinese medicine were the main therapeutic intervention for amblyopia in six of the included [11, 18, 19, 21, 32, 49].

#### Outcome measure domains

We identified ten domains of outcome measures in these studies (Table 1): “visual acuity” (86%), “stereopsis or sensory outcomes” (40%), “adverse events” (33%), “health-related quality of life” (HRQoL) (21%), “compliance” (19%), “refractive outcomes” (12%), “ocular alignment” (10%), “economic data” (7%), “visual evoked potential” (VEP) (5%), and “detection by photoscreeners” (2%).

**Table 1:** Amblyopia outcome measures and measurements

Outcome measure domain	Studies reported the outcome measure (n/42)	Variable subdomains of outcome measure	Variable outcome measurements	References
Visual acuity	36 (86%)	BCVA (median/ mean change,IOD,≥2 line improvement) 38 Uncorrected Distance Visual Acuity UDVA 1 Near Visual Acuity 1 Binocular VA 1	Age appropriate test 23 HOTV 7 ETDRS 5 Snellen eye chart 4 The Crowded Acuity Test (Glasgow Acuity Test) 2 Tumbling E charts 2 Bailey-Lovie 1 Allen Figs. 1	[3, 11–20, 22–34, 36–40, 43–48]
Stereopsis	17 (40%)	Near Stereopsis/ binocularity/sensory outcomes 16 Stereo sensitivity 1	Randot Preschool Stereoacuity Test 4 Lang stereo test II 2 Bagolini glasses response (at near & distance) 2 The Frisby test 2	[11, 12, 15, 20, 23–28, 31, 34, 36, 39, 42, 45, 48]
Adverse events	14 (33%)			[3, 11–13, 15, 16, 25, 27, 34, 35, 44, 47–49]
Health-related quality of life	9 (21%)	HRQoL 9 Emotional impact 1 Social stigma scores 1	Amblyopia Treatment Index 4 Amblyopia and Strabismus Q 2 Psychological Impact Q 2 Patching Success Q 1 CVFQ 1 SPPC 1 VF-14 1 Protection Motivation Theory Q 1 Interviews 1 Mean Rutter behaviour scores 1	[3, 11, 12, 15, 37, 41, 47, 48, 50]
Compliance	8 (19%)		Objective occlusion dose monitoring 3 Parental treatment diaries 2	[12, 15, 25, 27, 30, 34, 37, 44]
Refractive outcomes	5 (12%)	Median spherical equivalent 3 Refractive outcome 2 Spherical and cylindrical refraction 1	Manifest and cycloplegic refractions 1	[13, 14, 23, 24, 31]
Ocular alignment	5 (12%)		Simultaneous prism and Cover test SPCT 4 Cover test 1	[27, 31, 34, 42, 44]
Economic data	3 (7%)	Cost data 3 Cost- effectiveness 1		[3, 47, 48]
Visual evoked potentials	2 (5%)	M-VEP amplitude & latency 1 VEP latency 1		[16, 46]
Detection by Photoscreeners	1 (2%)		MTI photoscreener 1 PowerRefractor 1 PowerRefractor II 1 Plusoptix photoscreener series 1 iScreen 1 Spot 1	[1]

*BCVA*, Best Corrected Visual Acuity, *IOD* inter-ocular difference, *ETDRS* Electronic Early Treatment Diabetic Retinopathy Study, *UDVA* Uncorrected Distance Visual Acuity, *HR-QoL* Health-related quality of life, *Q* questionnaire, *CVFQ* Children’s Visual Function Questionnaire, *SPPC* Self-Perception Profile for Children, *VF-14* Visual Function Index, *VEP* Visual Evoked Potential, *M-VEP* multifocal VEP

#### Outcome measure subdomains and measurements

##### Visual acuity

The majority of studies (86%) measured visual acuity (VA) as the primary outcome measure. Variable descriptions used included improvement in VA [11, 18, 25, 28, 32, 35, 39, 45, 47], mean VA [3, 12, 13, 23, 40, 44, 48], median change in VA [23, 24], and “an increase of two or more lines of visual acuity or a final visual acuity of 20/25 or better” [20]. We identified a minority of subdomains of the outcome VA being reported by single studies such as near VA to compare it to distance visual acuity prior to amblyopia treatment [17] and “binocular VA” [39].

The LogMAR unit was universally used by all studies to report VA (*n* = 38) however different charts and distances were used depending on varying factors such as participant’s age or setting. Relative to studies that specified which charts were used, the most commonly reported tests were “Isolated Crowded Amblyopia Treatment Study HOTV for subjects aged 3 to < 7 years” [14, 17, 27, 34, 39, 44, 45] and “Electronic Early Treatment Diabetic Retinopathy Study VA protocol for subjects aged 7 or older” [14, 27, 34, 44, 45]. “Snellen chart” was reported as an alternative by a lesser number of studies [14, 16, 39, 45] and “Crowded Acuity Test” was used in two studies [30, 43].

##### Stereopsis/sensory outcomes

These were reported in 17/42 (40%) of the studies. In one study “stereo-sensitivity” was reported rather than stereopsis, in order to be able to represent nil stereoacuity by zero, which therefore can facilitate quantitative analysis as suggested by Tsirlin et al. [45].

Seven out of 17 of the studies did not report a particular outcome measurement, however the unit was given as “seconds of arc” in six studies [11, 12, 20, 23, 28, 42]. To measure near stereoacuity, “Randot Preschool test” was reported in four studies [27, 31, 34, 45], “Frisby test” in two [26, 45], and “Lang stereo test II” in two studies [23, 24]. “Bagolini glasses at distance & near” was used in addition, to determine lower levels of binocularity in the same previous two studies by Agervi et al. [23, 24].

##### Adverse events

The reported variants of this outcome measure included “diplopia” [12, 35, 47, 48], “occlusion amblyopia” [12, 47], “visual disorientation” [47], “skin irritation” [15], and “allergy to patches” [47, 48]. Adverse events were assessed using “a survey containing 17 items with a Likert scale completed by child and parent” in the RCT of Levodopa in older children by the Pediatric Eye Disease Investigator Group (PEDIG) [44]. The remaining studies did not give a particular method to gather this outcome measure.

##### HRQoL

This is increasingly being reported as an outcome measure in the treatment of amblyopia. The studies reported more than ten different instruments. The most commonly reported questionnaire in these was “The Amblyopia Treatment Index (ATI)” [37, 41, 50, 51].

##### Compliance

This was assessed using “objective occlusion dose monitoring” in three studies [12, 30, 37], by discussions with the parent [34], or review of a calendar log maintained by the participant and parent [44].

##### Ocular alignment

Interestingly ocular alignment was not reported as an outcome measure in the majority of the included studies (88%), even for strabismic amblyopia. However, it was highlighted in the PEDIG trials where it was measured using a “simultaneous prism and cover test” [27, 31, 34, 44] and in one Cochrane review where it was measured using “cover test” [42].

##### Refractive outcomes

“Median spherical equivalent” [14, 23, 24] and “spherical and cylindrical refraction” [13] have been reported in included studies.

##### Visual evoked potential (VEP)

VEP was reported as a secondary outcome in addition to visual acuity in the study conducted by Ivandic et al. after the use of low laser for adolescents and adults with amblyopia [46]. “Multifocal visual evoked potentials (M-VEP) amplitude and latency” was measured in a number of the subjects in the trial. Another example of using “VEP latency” as an outcome measure was reported by Yang et al. in a meta-analysis looking at studies that used Levodopa in the treatment of amblyopia in children < 18 years of age [16].

### Timing of measurements

We found variable timings that ranged from six weeks (post binocular training) [35, 40] to three years (post strabismus surgery in amblyopia [42], and post auricular point sticking therapy [18]. However 10 weeks [25, 27, 34], 6 months [11, 32, 40] and 12 months [3, 12, 29, 47, 48] were the commonest timings given. Long-term outcomes were measured at 15 years of age in the RCT of “Atropine vs patching for treatment of moderate amblyopia” by the PEDIG [31] and at seven years of age in the review of “Occlusion for stimulus deprivation amblyopia” [48].

### Strabismus

#### Types of included studies

We included 33 strabismus studies distributed as nine Cochrane reviews, four systematic reviews,13 controlled trials, and seven non-systematic reviews.

#### Types of strabismus and included age groups

This review included outcome measures extracted from studies investigating a wide range of strabismus types in different age groups.

While strabismus in general was under evaluation in around one third of the included studies (33%), intermittent exotropia by itself was the focus in more than one third (36%). This might be a reflection of the fact that intermittent exotropia is a common form of childhood exotropia [52]. Moreover, it is well established that it is one of the commonest worldwide constituting around 25% of all strabismus types [5].

On the other hand, esotropia was the target condition in only five studies (15%) with “Infantile esotropia” being the type in four of them [53–56] and “High AC/A ratio esotropia in teenagers” in one [57]. Three vertical strabismus studies were also among studies included in our review; two on dissociated vertical deviation (DVD) management [58, 59] and another on inferior oblique overaction [60].

The majority of subjects targeted in included strabismus studies were from the paediatric age group. In this review more than half of strabismus studies had children less than 18 years of age as participants compared to only 12% for adults [61–64]. The remaining studies were either generalised for adults and children [2, 4, 5, 58, 60, 65–68] or did not state a specific age group [52, 59, 69].

#### Types of treatment

Over half of the studies (52%) discussed outcome measures following surgical interventions for strabismus, most commonly muscle surgery (45%) [5, 42, 55, 56, 60, 61, 64–72] and less for botulinum toxin injection [2, 54]. Muscle surgery and botulinum toxin injection were both combined in one study [63]. In contrast, non-surgical or conservative treatments were evaluated in five of included studies (15%) [52, 57, 73–75]. Another 15% of studies involved both surgical and non-surgical interventions and reported treatment outcome measures following either method [4, 53, 58, 59, 76].

#### Outcome measure domains

We identified 14 domains of outcome measures for strabismus (Table 2). The four most commonly reported ones were “motor alignment” (79%), “binocularity” (64%), “adverse events” (61%), and “health-related quality of life” (48%). The less commonly reported outcome measures included “visual acuity” (24%), “control of deviation” (24%), “fusional vergence” (15%), “ocular movements” (9%) and “*AC/A ratio*” (3%).

**Table 2:** Strabismus outcome measures and measurements

Outcome measure domain	Studies reported the outcome measure (n/33)	Variable subdomains of outcome measure	Variable outcome measurements	References
Motor alignment	26 (79%)	Motor alignment / angle of deviation 26 Orthotropia or microtropia 1 Manifest strabismus 1 Hyperdeviation 1	Prism and alternate cover test 12 Simultaneous prism and cover test 8 Cover-uncover test 5 Synoptophore 4 Alternate cover test 3 Hirschberg test 3 Krimsky 3 Prism Bar Cover Test 1 Prism under cover test 1 Corneal reflection tests 1	[2, 4, 5, 42, 52–60, 63, 65–68, 70, 71, 74–79]
Binocularity	21 (64%)	Presence and quality of binocularity /binocular single vision 9 Stereoacuity at near 8 Steroeacuity at near & distance 4 Sensory fusion 2	Randot stereoacuity test 3 TNO 3 Titmus Housefly 3 Bagolini glasses 2 Worth’s 4 dot test 2 Frisbye Davis Distance (FD2) stereotest 1 Simultaneous perception 1 Suppression 1 Presence of monofixation 1	[2, 4, 5, 42, 52, 53, 55–60, 66–68, 71, 73, 75–77, 79]
Adverse events	20 (61%)	Induced A or V pattern 2 Induced vertical deviation 2 Development of DVD 2 Induced incomitance 2 Intolerable diplopia 2 Development of amblyopia 2 Induced ptosis 2 Long-term Change: re-operation rate/ recurrence /overcorrection/post-operative drift 2 Subconjunctival haemorrhage 1 Discomfort/abnormal sensory experiences 1	Tangent screen (in degrees) 1	[2, 4, 5, 42, 53–56, 58–61, 63, 65, 68–71, 76, 78]
Health-related quality of Life	16 (48%)	Improvement in quality of life 14 Patient satisfaction 2 Anxiety 1 Depression 1 Social anxiety and social avoidance 1 Well-being 1	Intermittent Exotropia Q 6 Amblyopia and Strabismus Q 4 Adult Strabismus Q 4 Age-specific QoL assessments 2 SF-36/ SF-8/ SF-12 2 EQ-5D 2 NEI-VFQ 2 VF-14 2 EYE-Q 2 Quality of life questionnaires 2 Vision-specific quality-of-life instruments 1 Any measure of patient or parent satisfaction relating to improvement to lifestyle 1 VFQ-25 1 The amblyopia treatment index 1 CVFQ 1 VQoL_CYP 1 CAT-QoL 1 PPQ 1 LVP-FVQ 1	[2, 4, 5, 50, 51, 53, 57, 58, 61, 62, 64, 65, 72, 73, 78, 79]
Visual acuity	8 (24%)	VA tests/BCVA 5 Amblyopia 3	Log MAR or log MAR equivalent 4 Snellen 1	[42, 52, 59, 63, 73, 77–79]
Control of deviation	8 (24%)	Control 7 Control of the near angle 1 Ability to maintain /control phoria with a filter 1 Control of DVD 1	Newcastle Control Score 5 Holmes and Mohney Office Control Scale 2 Mayo Score 2 Petrunak and Rao’s five-point Scale 1	[5, 52, 59, 68, 74, 76, 78, 79]
Fusional vergence	5 (15%)	Fusional vergence or amplitude for distance and near 3 Motor fusion test at near or distance or both 2	Base out or base in prism test/ Synoptophore 1 A prism bar 1	[2, 4, 42, 66, 68]
Economic outcomes	4 (12%)	Economics (e.g. length of stay in hospital, hours of surgeons time) 2 Use of health-care resources 2 NHS costs 2 Costs to families accessing the treatments 2		[2, 65, 78, 79]
Ocular movements	3 (9%)	Ocular movements 3 Inferior oblique function 1 DVD 1 DHD 1	Ordinal scale from 0 to 4+, grade 0 or 1+ is satisfactory 1 DVD grading scale of 1–4 1 DHD is measured by reversed fixation test 1	[54, 59, 60]
Re-operation rates	2 (6%)			[55, 65]
AC/A ratio	1 (3%)			[52]
Abnormal head posture	1 (3%)			[59]
Presence of latent nystagmus	1 (3%)		Video-oculography 1	[59]
Detection of strabismus using refraction devices	1 (3%)		Plusoptix Vision Screener 1	[77]

*TNO* The Netherland Organisation, *DVD* Dissociated Vertical Deviation, *DHD* Dissociated Horizontal deviation, *BCVA* Best Corrected Visual Acuity, *HR-QoL* Health-related quality of life, *Q* questionnaire, *SF* Short Form, *EQ5D* EuroQoL-5D, *EYE-Q* Effects of Youngsters’ Eyesight on Quality of Life, *CVFQ* Children’s Visual Function Questionnaire, *VQoL-CYP* Vision-related Quality of Life of Children and Young People, *CAT-QoL* Children’s Amblyopia Treatment Quality of Life Questionnaire, *PPQ* Perceived Psychosocial Questionnaire, *LVP-FVQ* LV Prasad–Functional Vision Questionnaire, *NEI-VFQ* National Eye Institute Visual Function Questionnaire, *VF-14* Visual Function Index, *NHS* National Health Service

#### Outcome measure subdomains and measurements

##### Motor alignment/angle of deviation

This was reported as “motor alignment” or “angle of deviation” in 26/33 studies. This was further described to be measured “at near and distance” in seven studies out of these [4, 5, 63, 66, 70, 71, 77].

In 12 studies alignment was measured using “prism alternate cover test PACT” [4, 5, 54, 58, 59, 63, 67, 70, 71, 74, 77, 78] and /or with “simultaneous prism cover test SPCT” in eight studies [4, 53, 63, 66, 74, 75, 77, 79]. “Cover test” was reported in five studies [2, 42, 73, 74, 79], “Synoptophore” in four [2, 4, 42, 53] and “Hirschberg test” in three [54, 73, 79]. Krimsky test was reported as an alternative test in subjects with poor cooperation in one study [54], when cover tests are not applicable [63] or in cases with poor vision (worse than 20/200) in one RCT [60].

It is noteworthy that there is still no total agreement on the definition of a successful ocular alignment [5], varying from 5 to 8 to 10 PD from orthophoria. However there was a considerable agreement on defining success in included studies as orthophoria within 10 PD [2, 42, 53, 61, 65, 74, 80].

##### Stereopsis/sensory outcomes

Sensory outcomes were either reported as any level of “binocularity/stereopsis” [2, 52, 55, 57–59, 67], or as “stereoacuity” (near or presumably near) [5, 56, 60, 71, 73, 76, 77, 79], or both; with “binocularity” and “stereoacuity” stated as two distinct outcome measures [42]. Additionally, “steroacuity at near and distance” was measured in four of intermittent exotropia studies [4, 66, 68, 74].

The outcome measurement used to assess stereoacuity were similar to those found in amblyopia studies, “Randot stereoacuity test” [71, 73, 74], with the addition of Titmus Housefly [42, 55, 60] and TNO [52, 66, 67]. “Sensory fusion” was measured with “Worth’s 4 dots” test in two studies [42, 67]. In one review, a stepwise approach of assessing binocularity was undertaken. After “stereoacuity” (the gold standard), “simultaneous perception” and “motor fusion” were considered next [53].

##### Adverse events

These included postoperative alignment complications such as “induced A or V pattern” [60, 70], “induced vertical deviation” [2, 54] “development of DVD” [56, 60], “induced incomitance” [69, 71], or visual complications such as intolerable diplopia [2, 65] and “development of amblyopia” [4, 53].

Other adverse events specified in included studies were “globe perforation” [65, 70, 78], and “induced ptosis” post botulinum toxin injection [2, 53, 54].

Long-term change following surgical procedures including “recurrence of deviation” [68], “overcorrection” [4, 78] and “re-operation rate/number of operations needed” [53, 55, 65, 78] were reported.

##### HRQoL

This was assessed using different questionnaires that could be generic (for example SF-36) [61, 62], or specific to age (for example EYE-Q) [77, 78], specific to vision (for example VFQ-25) [58] or specific to condition (for example the IXTQ) [5, 50, 51, 62, 72, 77].

Strabismus and amblyopia were often combined in the same HRQoL questionnaire (for example the A&SQ) [50, 61, 62, 64].

##### Visual acuity

Only 24% of included strabismus studies reported BCVA as an outcome measure. LogMAR or LogMAR equivalent was the most reported unit used [42, 52, 63, 79].

##### Control of deviation

This outcome measure was reported in seven intermittent exotropia studies [5, 52, 68, 74, 76–78]. Different scores were used including “Newcastle Control Score” [5, 52, 68, 77, 78], “Office Control Score” [5, 74], “Mayo Score” [77, 78] and “Petrunak and Rao’s five point scale” [52]. “Control to show whether the deviation is latent or manifest” was also considered for DVD in the review by Christoff et al. [59].

##### Fusional vergence

A further outcome was referred to as “fusional vergence for distance and near” in three [2, 66, 68] or as “motor fusion at distance or near or both” in two studies [4, 42]. It was measured in one included study using a “base out or base in prism test/synoptophore” [42] or “a prism bar” [66].

##### Ocular movements

These were included in vertical strabismus such as DVD [59] and inferior oblique overaction [60]. Muscle action was documented on a grading scale from 1 to 4 [59] or 0–4 [60]_._

##### AC/a ratio

AC/A ratio was reported as an outcome in a review by Piano et al. for the conservative treatment of intermittent distance exotropia [52].

#### Timing of measurements

The time of measurement varied between studies and did not clearly correlate with the intervention. The measurement was often done at multiple time points [54, 61, 63, 66, 67, 70, 78] or at one time point otherwise. It ranged from one week [66] to three years [42, 73]. The most frequently given timings were 3 months [54, 60, 61, 63, 66, 69, 70, 72, 78], 6 months [2, 53, 54, 61, 63, 65–67, 70, 71, 74–76, 78] and one year [58, 61, 63, 64, 67]. Long-term outcomes were measured at age of six years in one study for infantile esotropia [55].

### Ocular motility disorders (OMDs)

#### Types of included studies

A total of 68 studies were included for ocular motility disorders (OMDs), distributed as eight Cochrane reviews, 12 systematic reviews, 29 controlled trials, 15 non-systematic reviews and four retrospective studies.

#### Types of ocular motility disorders and included age groups

We classified OMDs into seven sub-conditions. Table 3 gives an outline of the common outcome measures across these sub-conditions in included studies.

**Table 3:** Ocular motility disorder sub-conditions and common outcome measures

OMD sub- condition	Range of eye movement	HRQoL	Diplopia	VA	Motor alignment	Adverse events	AHP	Patient symptoms	BSV	Stereopsis
Accommodation & convergence disorders		√		√	√			√		
Mechanical & paralytic	√	√	√	√	√	√	√			
Myogenic	√	√	√	√	√	√	√	√	√	√
Neurogenic	√		√	√	√	√	√		√	
Nystagmus	√	√		√		√	√			
Patterns	√				√	√				√
Central causes	√		√		√		√	√	√	√

*OMD* ocular motility disorder, *HRQoL* Health-related quality of life, *VA* visual acuity, *AHP* abnormal head posture, *BSV* binocular singe vision

Forty five per cent of included OMDs studies [81–116] had adults exclusively as subjects due to the nature of conditions under evaluation such as thyroid eye disease and neurological diseases with gaze palsies, which exist in adults typically. In contrast, less than tenth of the studies were done on paediatric subjects [117–124]. Some of these were for conditions found predominantly in teenagers such as convergence insufficiency and accommodation dysfunction and others included disorders with an early onset such as infantile nystagmus and pattern deviations. The remaining studies had mixed adults and children populations (*n* = 18/67) [80, 125–141] or the age was not clear (*n* = 6/67) [142–147].

#### Types of treatment

Interventions used in these studies included medical, surgical and conservative measures.

#### Outcome measure domains

We identified ten domains of outcome measures common for the majority of OMDs (Table 4). The most frequently reported outcome measures were “range of eye movement” (34%), “HRQoL” (28%), “improvement in diplopia” (26%), “visual acuity” (22%) and “motor alignment” (21%). Other less frequent outcome measures included “adverse events” (15%), “improvement in symptoms” (13%), “improvement in AHP” (10%), “increasing field of BSV” (6%) and “stereoacuity” (6%).

**Table 4:** Ocular motility disorder outcome measures and measurements

Outcome measure domain	Studies reported the outcome measure (n/68)	Variable subdomains of outcome measure	Variable outcome measurements	References
Range of eye movement	23 (34%)	Range of eye movement/Change in extraocular motility 14 Determination of range of motility 4 Oculomotor range/amount of ductions 4 Restriction of eye movements 3 Motility assessment 3 Quantitative measurement of eye movement 1 Assessment of ocular muscle contracture 1	Gradation of movements 2 A scale of − 4 underaction to + 4 overaction, with 0 being normal 2 Forced duction test 2 The CROM score 2 A and V patterns 1 Two-step test 1 Monocular (ductions) and binocular (versions) 1 Cover/uncover test 1 Videotaped, measured the nine positions of gaze directly on photographs 1 Downshoot 1	[80–82, 89, 90, 92, 93, 95, 102, 108, 111, 115, 116, 119, 121, 129, 130, 132, 134, 138, 139, 141, 144]
Health-related quality of Life	19 (28%)	Improvement in HRQoL 16 Patient satisfaction and functional measurements 2 Participant and physician- reported global health assessment 1 Aesthetic outcome 1 Appearance of the eye 1 Functional outcome 1 Being able to drive after strabismus surgery 1	Validated questionnaires 6 Short Form 36 (SF-36) 2 Sickness Index Profile (SIP) 1 Visual analogue scale 1 NEI-VFQ-25 3 Visual analogue scale 1	[80, 82, 84, 87, 91, 95–97, 104, 107, 109–111, 115, 116, 125, 126, 142, 143]
Improvement in diplopia	18 (26%)	Recovery in diplopia 18 Disappearance of diplopia in primary gaze 2 No diplopia in primary position and downgaze with prisms or without prisms 1	Subjective diplopia score (Gorman scale) 5 Diplopia score within GO-QOL questionnaire 1 A field diplopia test 1	[89, 93, 101, 105, 106, 108, 111, 112, 114–116, 119, 127, 133, 134, 141, 144, 146]
Visual acuity	15 (22%)	Visual Acuity/BCVA 11 Binocular BCVA 5 Near VA 1 Estimated VA 1	Log MAR or Snellen 4 Snellen 1 Pattern reversal VEP 1 Subjective score within GO-QoL questionnaire 1	[85–87, 96–98, 101, 108, 112, 119, 120, 124, 126, 145, 147]
Motor alignment	14 (21%)	Deviation 4 Objective torsion 4 Ocular alignment testing 2 Phoria 2 Alignment in primary position 1 Incomitance 1 A & V pattern 1 Pattern deviation and horizontal deviation 2 Subjective torsion 1	PACT 2 Indirect ophthalmoscopy 2 Cover tests 1 PCT 1 Krimsky 1 Synoptophore 1 Maddox rod to test overcorrection > 20 degrees 1 Double Maddox rod test 1	[106, 110, 121, 124, 128, 130–132, 134, 136, 138–140, 147]
Adverse events	10 (15%)	Related to steroids 2 Related to Rituximab 1 Related to radiotherapy 1 Related to acupuncture 1 Surgical complications 1 Vision loss/retro orbital hematoma post reconstruction 1 Surgery-induced strabismus or visual loss 1 Post op drift 1		[82, 84, 93, 99, 111, 126, 127, 130, 136, 146]
Improvement in symptoms	9 (13%)	CI symptoms 5 Ocular myasthenia symptoms 1 Oscillopsia or blur in nystagmus 1 Patient-reported symptoms post brain injury 1	**(**CISS) Version-15/CISS score 6 Patient record or notes/questionnaire 1	[80, 117, 123–125, 127, 128, 137, 147]
Improvement in abnormal head posture	7 (10%)		(In degrees) 2 Inspection 1	[106, 120, 126, 129, 131, 132, 134]
Increasing the field of binocular single vision (BSV)	4 (6%)		Goldman perimeter with the score system of Sullivan 1 Worth four dot test 1 CROM device 1	[80, 110, 130, 134]
Stereopsis	4 (6%)	Presence of stereopsis 2 Steroacuity 2	TNO stereo test 1	[80, 106, 124, 140]

*HR-QoL* Health-related quality of life, *Q* questionnaire, *CROM* cervical range of motion, *NEI-VFQ* National Eye Institute Visual Function Questionnaire, *SF* Short Form, *BCVA* Best Corrected Visual Acuity, *VEP* Visual Evoked Potential, *GO-QoL* Graves Ophthalmopathy Quality of Life, *APCT* Alternate Prism Cover Test, *PCT* Prism Cover Test, *TED* Thyroid Eye Disease, *CISS* Convergence Insufficiency Symptom Survey, *TNO* The Netherland Organisation

#### Outcome measure subdomains and measurements

##### Range of eye movement

This was the commonest outcome measure reported in general for OMDs and was included in all sub-conditions except for accommodation and convergence disorders. This was either included in composite scores or as a distinct outcome measure. In one RCT the nine positions of gaze were videotaped and measurements were done “directly on photographs drawing a horizontal straight line from internal canthus” [102]. In another study this was described as “in 8 positions of gaze binocularly and monocularly” [132].

##### HRQoL

HRQoL outcome measures were mostly prominent in thyroid eye disease studies [82, 84, 91, 95–97, 104, 107, 109–111, 115, 116], nevertheless they were also scattered in other sub-conditions; accommodation and convergence disorders [125], ocular myasthenia [142] and central causes of eye movement disorders [80, 81].

##### Improvement in diplopia

This was another common outcome measure; however there was incongruity in the position of gaze free from diplopia. Position of gaze was mostly not indicated [90, 93, 101, 105, 112, 115, 116, 127, 133, 141, 144, 146], however improvement was confined to primary gaze in a number of studies [106, 108, 114, 119].

##### Visual acuity

Only 22% of the included studies reported visual acuity and these were mostly for nystagmus or orbital abnormalities. “Binocular visual acuity” was specifically additionally indicated in two of nystagmus studies [85, 126].

##### Motor alignment

This was reported in all sub-conditions except nystagmus studies. Whenever reported, this was either assessed with cover/uncover/alternate cover test without quantification [129, 132] or quantified using “PACT” [138, 140] or “Krimsky” [138] in less cooperative patients. Moreover, in addition to horizontal and vertical deviation, torsion was evaluated in a number of pattern deviation studies objectively [121, 136, 139, 140] or less commonly subjectively [121].

#### Timing of measurements

Multiple time points [82, 92, 94, 96, 97, 100, 101, 103, 115, 118, 121, 128] or spans of follow up were often given [83, 84, 99, 105, 111, 113, 126, 139]. However 6 months [82, 83, 97, 99, 103, 104, 111] and 12 months [82, 97, 98, 103, 107, 113] were frequent timings given in thyroid eye disease studies. Twelve weeks timing was common for accommodation and convergence disorders [117, 118, 125, 128].

More details on included studies for amblyopia, strabismus and ocular motility disorders arranged alongside identified outcome measures, outcome measurements and timings are given in Additional file 3: Table S31, Additional file 4: Table S3.2, Additional file 5:: Table S3.3 and Additional file 6: Tables S4.1–4.7).

### Outcome measures per sub-condition

#### Accommodation and convergence disorders (*n* = 7) (Additional file 6: Table S4.1)

For this group of disorders, the most prominent outcome measures were “patient symptoms” recorded with “Convergence Insufficiency Symptom Survey (CISS)” (86%). “Near point of convergence NPC” and “positive fusional vergence” were less common. Alignment measurement was not expansively assessed in included studies apart from measuring “phoria” in two studies [128, 147] or ruling out manifest strabismus with “cover test at distance and near” for inclusion in one trial [124] . **“**Amplitude of accommodation” [118, 128, 147], and “accommodative facility” [118, 147] were also reported. “Dynamic retinoscopy” was reported by one study [147].

#### Ocular mechanical restriction (*n* = 6) (Additional file 6: Table S4.2)

The outcome measures “resolution of diplopia”, “motility assessment” and “alignment” were mutual with other OMDs.

However, in certain circumstances such as in acute orbital floor fractures, the outcome measures “oculocardiac reflex” [119],“visual acuity” [119, 141] and “pupillary function” [119] were important.

“Assessment of fractures and entrapment of soft tissue” was evaluated with radiographic imaging such as helical CT [119].

“Forced duction test” was reported to check muscle restriction and entrapment in two included studies [119, 122]. Other assessments done in orbital fractures included “globe integrity” [119], “globe dystopia” [119, 122] and “infraorbital hyposthaesia” [105, 119, 141]. A further outcome measure related to appearance was “resolution of enophthalmos” [105, 141, 146].

#### Ocular myogenic disorders (*n* = 30) (Additional file 6: Table S4.3)

##### Thyroid eye disease

Treatment response in thyroid eye disease is commonly evaluated using composite scores such as *“*VISA” [109] and “EUGOGO score” [91, 92, 103]. Modified versions of existing scales are often developed and used (for example “modified EUGOGO” [101], and “modified Werner grading scale” for orbital inflammation [96]). In addition, we found a high frequency of a number of widely recognised scoring systems such as “The clinical activity score (CAS)” to assess disease activity [82, 84, 90, 92, 95, 97–99, 101, 103, 104, 108, 110–112, 114–116] and “NO SPECS” to assess disease severity. [82, 84, 90, 103, 104, 111]*.*

“Subjective diplopia” was frequently assessed using “the Gorman diplopia score” [90, 95, 116]. Ocular muscle motility assessment was mostly involved within composite scores but occasionally measured with dedicated scores (for e.g. The Total Motility score (TMS)) [116]. Additional outcome measures reported by studies for thyroid eye disease included “the need for post treatment corrective procedures” [83, 84, 91, 98, 111], and “orbital volume/orbital fat and muscle volume”. [95, 112].

##### Ocular myasthenia gravis and progressive external ophthalmoplegia

In addition to the previously stated outcome measures shared with other eye motility disorders such as “improvement in diplopia” [127, 133] and “eye movement measurement” [102], there were outcome measures specific to myasthenia reported by included studies. These included “quantitative ocular myasthenia gravis score (OMG) score” [142] and “progression to generalised myasthenia gravis” [127, 133, 142]. Other associated ocular motility abnormalities reported included “inter-saccadic fatigue”, “gaze-paretic nystagmus”, “fatigue of accommodation” and “reduced velocity of pupillary constriction” [133]. Quality of life was evaluated using “the 15-item Myasthenia Gravis quality of life scale” in one study [142].

#### Ocular motility disorders secondary to neurogenic disorders (*n* = 6) (Supp. Table 4.4)

These refer to conditions such as third, fourth and sixth cranial nerve palsies. Clinical outcome measures included here in addition were “palpebral fissure size” [93, 144] and “pupil size” [144] for third nerve palsy. Bi et al. used “The cervical range motion (CROM) score” to quantify diplopia in a pilot RCT on acupuncture for the treatment of oculomotor paralysis [93].

In the review by Engel, in congenital fourth nerve palsy, alignment was checked with “a more sensitive 2-step test” [131]. “Facial asymmetry” was evaluated, and “superior oblique muscle atrophy/absent trochlear nerve” were examined with “high definition MRI” in the same study [131]. “Abnormal head position” was measured objectively using “a goniometer” in degrees [131]. An important adverse event sought after treatment here included “secondary Brown syndrome” [131].

In sixth nerve palsy, motility outcomes were included to reveal any “degree of incomitance” while measuring deviation, and to check for “medial rectus muscle contracture” using “forced duction test” [130]. “Scott’s force generation test” or “electrooculography/electromyography” were used to assess “lateral rectus muscle function” [130].

#### Nystagmus (*n* = 8) (Additional file 6: Table S4.5)

Outcome measures that were shared with most of the remaining OMDs were “visual acuity” (75%) [85–87, 120, 126, 145], “improved head posture” (25%) [120, 126], “patient satisfaction” (25%) [87, 126] and “range of eye movement” (13%) [132]. However, it is important to note that vision in patients with nystagmus was assessed more comprehensively with additional specifications in a few studies; “binocular visual acuity” was reported in two studies [85, 126], “gaze-dependant visual acuity GDVA” in one study [86], “near visual acuity” in one study [87] and “estimated visual acuity using pattern reversal VEP” in one study for infantile nystagmus [126].

“Eye movement recordings” was included in six nystagmus studies (75%) [85–87, 126, 132, 145]. Examples of the methods used to record eye movement included “3-D video-oculography” [87] and “an infrared video pupil tracker” [86]. Different specific characteristics of nystagmus were gathered from eye movement recordings including “foveation/recognition time” [126, 137], “broadening the null point” [137, 145], and “nystagmus waveform” [126, 145]. The specific symptom of “oscillopsia” was assessed in two included studies [120, 137].

#### Pattern deviation (*n* = 5) (Additional file 6: Table S4.6)

A special feature with this group of conditions was the torsion deviation measurement reported in 80% (*n* = 4/5) of studies [121, 136, 139, 140]. “Objective torsion” using “indirect ophthalmoscopy” was more commonly reported [121, 136, 139, 140] than “subjective torsion using double Maddox rod test” [121]. An example of a grading scale used was “-4 underaction to +4 overaction with 0 being normal” [121].

#### Ocular motility disorders secondary to central causes (*n* = 7) (Additional file 6: Table S4.7)

These include gaze palsies and some forms of acquired nystagmus. In addition to the common outcome measures with other sub-conditions, there were others highlighted in a number of included studies. These comprised particular attention to “saccades and pursuits”. Measurements were done using “the optokinetic drum” or “video-oculogarphy” [132]. “Near point of convergence” was reported in one study [106].

## Discussion

Systematic reviews investigating various specialities including ophthalmology [148–151] are increasingly being performed. What is evident from many systematic reviews is that the results from included trials and studies cannot be meta-analysed because of the variation in outcome measures used across the studies. The COMET initiative calls for development of COS in order to provide a minimum set of outcome measures which will facilitate future synthesis of results. To our knowledge this is the first review using systematic methods in accordance with the COMET handbook aiming to develop an item bank of outcome measures in the treatment of amblyopia, strabismus and ocular motility disorders.

We chose to combine these conditions in one report due to the great overlap between them and their frequent co-existence in subjects. Indeed some might consider strabismus as a subset of ocular motility disorders and vice versa. For example esotropia from sixth cranial nerve palsy was classified under motility disorders while others may classify it under strabismus. Additionally, strabismus can cause or result from amblyopia, and similarly with ocular motility disorders with childhood onset. Therefore it is meaningful to consider them all in one generalised report.

Although we did not cover every type, this review includes outcome measures extracted from studies investigating a wide range of amblyopia, strabismus and ocular motility disorders in different age groups undergoing nearly all possible methods of interventions.

### Amblyopia

Although we attempted to include all types of amblyopia in this paper, we found that the majority of the studied variants were anisometropic, strabismic and combined anisometropic and strabismic amblyopia. Even though aetiologies were different, therapeutic interventions and outcome measures were comparable.

This review found that VA is the only outcome measure agreed by the great majority of included amblyopia studies. Stereopsis, adverse events and HRQoL were also relatively common however they were reported by less than half of the studies. VA and stereopsis measurement methods largely depended on the age of subjects who were mostly from the paediatric age group.

BCVA is measured typically in children from around the age of 3–4 years as well as in adults. It is the most commonly used outcome to assess visual acuity in our review and in perhaps in general for any eye condition. However, it is increasingly recognised that it does not truly reflect visual function needed in normal daily activities [151]. Additional assessments that can give more information about visual function include contrast sensitivity, near visual acuity, reading speed and visual field sensitivity [151].

It is not uncommon to find older children and adults with residual amblyopia, and as a result various non-conventional therapies attempted to treat it beyond the plasticity period. When that is done visual function can be assessed using conventional methods in addition to more objective and sensitive methods especially in the research environment. VEP is one outcome measure used to assess visual function post treatment in older children and adults. It is recommended to use VEP latency rather than amplitude due to its higher sensitivity [46].

Due to the strong association between amblyopia and strabismus, we made the assumption that ocular alignment would be a standard outcome measure in amblyopia studies, which was not the case once results had been gathered and analysed. Only 12% of the studies included this outcome measure.

Regarding health-related quality of life, it is notable that treatment side effects and compliance are occasionally evaluated and reported within HRQoL questionnaires, i.e. collecting all subjective or patient-reported outcomes in one type of a composite score. Therefore a number of amblyopia studies that reported HRQoL did not consider adverse events or compliance as independent outcome measures.

The timing of reported measurements was variable between studies however the most frequent time point found here was 12 months.

### Strabismus

There is nearly a total agreement on the necessity to measure motor alignment at distance and near using prism alternate cover test (PACT) or simultaneous prism cover test (SPCT) in ideal situations; and Krimsky in poor cooperation or low vision [60]. The difference between PACT and SPCT is that the first measures the alignment by covering each eye alternatively whereas the second measures alignment before binocular vision is disrupted. Generally the total misalignment measured by PACT is the most often one reported [77].

The other outcome measures reported by more than half of strabismus studies were “binocularity (stereopsis/BSV)” and “adverse events”. “HRQoL” was reported by just under half of included studies.

Binocularity was mostly measured in included studies using near stereopsis. We found that distance stereopsis is not typically assessed with the exception of intermittent exotropia. A moderate correlation was found between near and distance stereoacuity in previous studies [66] and most clinicians prefer to measure near stereoacuity over distance stereoacuity because of better patient cooperation [5]. On the other hand, some authors suggest that distance stereoacuity is a better indicator for intermittent exotropia progression [66]. For example, in the RCT conducted by Saxena et al., distance stereoacuity showed continued improvement for up to three months post treatment compared to one week for near stereoacuity [66].

HRQoL is a complex concept with wide variation in how people perceive it individually and within one individual over time [62]. There is no agreed definition of QoL [51] however it can be considered a reflection of one’s overall well-being and life experience, which is affected by different factors including physical, psychosocial and environmental elements [62]. McBain et al. found that adults with strabismus can have one of two types of QoL concerns; for example there may be functional concerns for those with diplopia and psychosocial concerns for those with strabismus but no diplopia [62]. It must be highlighted that the aim of measuring HRQoL outcome is to provide appropriate support depending on specific concerns or needs. It seems nevertheless that there is still no total consensus on one method of measurement of HRQoL in strabismus and amblyopia and that there is room for further development to reach agreement.

In comparison to the agreement on the above measures, there was dissimilarity in measuring other outcome measures such as “visual acuity” and “control of deviation” for patients with strabismus.

This review found only one third of strabismus studies considering “visual acuity” important to measure after treatment. This could be partially explained by the fact that it is relevant mostly in children to check the status of amblyopia and that vision is not a primary concern when there is prior amblyopia in adults undergoing for example surgical correction.

Furthermore, there are a number of outcome measures relevant only in specific variants of strabismus for example “control of deviation” and “AC/A ratio”. Control of deviation is pertinent mostly in cases of intermittent exotropia and DVD. AC/A ratio is important mostly in high AC/A esotropia.

“AC/A ratio” is often measured for intermittent exotropia as well. It was shown previously by some authors that lower AC/A ratios were attained post extensive orthoptic exercises for intermittent distance exotropia [52]. However, due to technical difficulties in measurements and potential inaccuracies if occlusion is not used while measuring it to differentiate between true and pseudo divergence excess, it is challenging to use it as a standard test to guide treatment [52].

Six months was the most commonly given timing to report outcome measures post strabismus treatment although there was great variation between studies.

### Ocular motility disorders

Agreement on outcome measures for OMDs was the least compared to amblyopia and strabismus probably due to the wider variation in clinical features and therefore we provided outcome measures per sub-condition. However we found a degree of overlap in some outcome measures between the seven categories such as “range of eye movement”, “HRQoL” and “improvement in diplopia”.

Generally, it seems that having a satisfactory “range of eye movement” was the preferred outcome measure in eye motility disorders and that measurement in both ductions and versions is recommended to differentiate restrictive from paralytic eye conditions.

“HRQoL” assessment was shown to be especially relevant in disfiguring conditions such as thyroid eye disease. The reason behind that is the previously noted psychological factors which do not correlate well with objective clinical measures for unclear reasons [107]. There have been various versions of Graves’s ophthalmopathy QoL questionnaires, but once more there is no consensus regarding their use [109]. A common feature in such questionnaires however is addressing both visual and appearance-related aspects of QoL [97, 107, 110]. Some authors considered in addition evaluating long-term quality of life in this group of patients for up to 11 years [107].

Furthermore, for OMDs complicated with “diplopia”, a primary outcome measure frequently emphasised here was to assess improvement or resolution of diplopia. However, it would be useful, we suggest, to have an agreement whether any improvement in diplopia would be acceptable or improvement in diplopia in primary gaze, down gaze, with or without prisms would be required to define success. Also whether subjective reports are sufficient or they need to be combined with objective measurement of “field of binocular single vision”. Similarly for measurement of deviation or reporting “alignment”, an indication whether orthophoria in primary gaze or in more positions of gaze to be planned or achieved would be more helpful.

“Improvement in head posture” was found often closely related to improvement in diplopia and alignment, however this review has shown that it was not consistently addressed in relevant studies. Reporting head posture improvement in relation to the null position was similarly incongruous in nystagmus studies.

On the other hand, when diplopia was not the only concern in the ocular motility disorder as in accommodation and convergence disorders, “improvement in symptoms” would be reported. “The Convergence Insufficiency Symptom Survey” appeared to be widely accepted for this purpose [117, 123, 125, 128, 147].

Although assessment of “visual acuity” is typically standard in eye conditions, it was not reported in 75% of included OMDs studies. As noted above, its measurement was shown to be vital in nystagmus patients mostly. However, consensus is needed about what category of visual acuity to measure. Vision assessment was also relevant in thyroid eye disease and orbital fracture for optic nerve function assessment in relation to orbital changes.

Timing of reported outcome measures here was variable due to various factors indicated above.

#### Study strengths and limitations

The strength of this work is that the review followed a prescribed process for the creation of an item bank of outcome measures [7]. The resultant item bank is a comprehensive list that underpins the first stage of the process to develop Core Outcome Sets for amblyopia, strabismus and ocular motility disorders.

On the other hand, despite some overlap between target conditions, the varied review scope and inclusion of a wide range of conditions together could be considered a limitation preventing us from finding all the relevant reported outcome measures for every target condition and sub-condition. Although generalised and overlapping outcome measures for amblyopia, strabismus and ocular motility disorders were extracted here, specific and more refined categories of outcome measures might have been overlooked.

Another potential limitation is the exclusion of other studies of lower quality than systematic reviews and controlled trials, which might have resulted in missing valuable sources of reported outcome measures in literature. It would not be possible however to include all types of studies for a wide group of conditions as in our review. This might be feasible for conditions/sub-conditions when investigated individually.

#### Future work and recommendations

We next plan to conduct an iterative consensus process (Delphi surveys and group meetings) with key stakeholders including patients, clinicians and researchers as the second stage of developing these COSs. This stage will be to standardise what to measure, i.e. outcome measures. Subsequent work will be required to standardise how to measure them, i.e. outcome measurements and later, when to measure them, i.e. timing of measurements.

In terms of developing “Core Outcome Sets”, we suggest the inclusion of both subjective and objective outcome measures; and both positive (i.e. improvement from baseline) and serious negative outcomes (i.e. adverse events). Furthermore, choosing feasible and easily available assessments is important. We also recommend that “long-term outcomes”, especially for known chronic conditions, are considered.

## Conclusions

We generated lists of the most reported outcome measures for amblyopia, strabismus and ocular motility disorders within included studies with indications to specific outcome measures in certain sub-conditions. We also identified the most reported outcome measurements and their timings from intervention to some extent.

This review also demonstrates significant variation in outcome measure reporting within published studies in the three conditions confirming the challenge in efficient comparison, combination and synthesis of data.

Various factors might be responsible for inconsistency between studies in reported outcome measures in conditions targeted in this review including age group, type of condition and often researcher or clinician preferences. While some of these factors are understandably fixed, researchers and clinicians preferences can probably be unified and standardised.

Although common outcome measures and measurements from the literature are highlighted in this review, this does not imply that they are necessarily the most appropriate outcome measures to be used as “core outcome measures” in trials or clinical practice. Consensus among all stakeholders including patients, clinicians, and researchers is required to establish COS. International agreement would be ideal to maximise usefulness of research overall.

## Additional files

Additional file 1: **Table S1.** PRISMA checklist. This table contains the different sections in the review with the items and the page(s) where these can be found. (DOC 66 kb)

Additional file 2: **Table S2.** Search terms of SCOPUS database. Terms and Boolean operators used to perform the search for the review in one example database- SCOPUS. (DOCX 19 kb)

Additional file 3: **Table S3.1.** Amblyopia included studies. Included studies for amblyopia arranged by type of study, study ID, title, outcome measure domain, outcome measurement and time of measurement. (DOCX 30 kb)

Additional file 4: **Table S3.2.** Strabismus included studies. Included studies for strabismus arranged by type of study, study ID, title, outcome measure domain, outcome measurement and time of measurement. (DOCX 34 kb)

Additional file 5: **Table S3.3.** Ocular motility disorders included studies. Included studies for ocular motility disorders arranged by sub-condition, study ID, title, outcome measure domain, outcome measurement and time of measurement. (DOCX 50 kb)

Additional file 6: **Tables S4.1-4.7.** Outcome measures per sub- condition of ocular motility disorder, Reported outcome measures arranged alongside OMD sub-conditions, number of studies reporting outcome measures, outcome measurements and references. (DOCX 194 kb)

## Abbreviations

A&SQ: Amblyopia and Strabismus Questionnaire
AC/A ratio: Accommodative convergence/Accommodation ratio
AHP: Abnormal Head Posture
BCVA: Best Corrected Visual Acuity
BSV: Binocular Singe Vision
CAS: Clinical Activity Score
CCT: Controlled clinical trials
CISS: Convergence Insufficiency Symptom Survey
COMET: Core Outcome Measures in Effectiveness Trials
COS: Core Outcome Set
CROM: Cervical Range Of Motion
CT: Computerised Tomography
DHD: Dissociated Horizontal Deviation
DVD: Dissociated Vertical Deviation
ETDRS: Electronic Early Treatment Diabetic Retinopathy Study
EUGOGO score: EUropean Group On Graves’ Orbitopathy
GDVA: Gaze-dependent Visual Acuity
GO-QoL: Graves Ophthalmopathy Quality of Life
HRQoL: Health-Related Quality of Life
IXTQ: Intermittent Exotropia Questionnaire
Log MAR: Logarithm of the Minimum Angle of Resolution
M-VEP: Multifocal Visual Evoked Potentials
NO SPECS: No Signs or symptoms, Only signs, Soft tissue involvement, Proptosis, Extraocular muscle involvement, Corneal involvement, Sight loss
OMDs: Ocular motility disorders
OMG: Ocular Myasthenia Gravis
PACT: Prism Alternate Cover Test
PD: Prism Dioptre
PEDIG: Pediatric Eye Disease Investigator Group
PROMS: Patient-reported outcome measures
RCT: Randomised Controlled Trials
SPCT: Simultaneous Prism Cover Test
TED: Thyroid Eye Disease
TES: Total Eye Score
TMS: Total Motility Score
TNO: The Netherland Organisation
UDVA: Uncorrected Distance VA
VISA: Vision, Inflammation, Strabismus/restriction, and Appearance/exposure
