# Correction to: development of a core outcome set for amblyopia, strabismus and ocular motility disorders: a review to identify outcome measures

**DOI:** 10.1186/s12886-019-1073-6

**Published:** 2019-03-11

**Authors:** Samia Al Jabri, Jamie Kirkham, Fiona J. Rowe

**Affiliations:** 1 Department of Health Services Research, University of Liverpool, Waterhouse Building Block B, 2nd Floor, 1-3 Brownlow Street, L69 3GL, Liverpool, UK; 2 Department of Biostatistics, University of Liverpool, Liverpool, UK

**Correction to: BMC Ophthalmology 2019 19:47**

**https://doi.org/10.1186/s12886-019-1055-8**

Following publication of the original article [1], the authors notified us that Fig. 3 was published as a table, when it should actually be a diagram of study countries. In this correction article, Fig. 3 was updated. The original article was corrected.

**Fig. 3:**
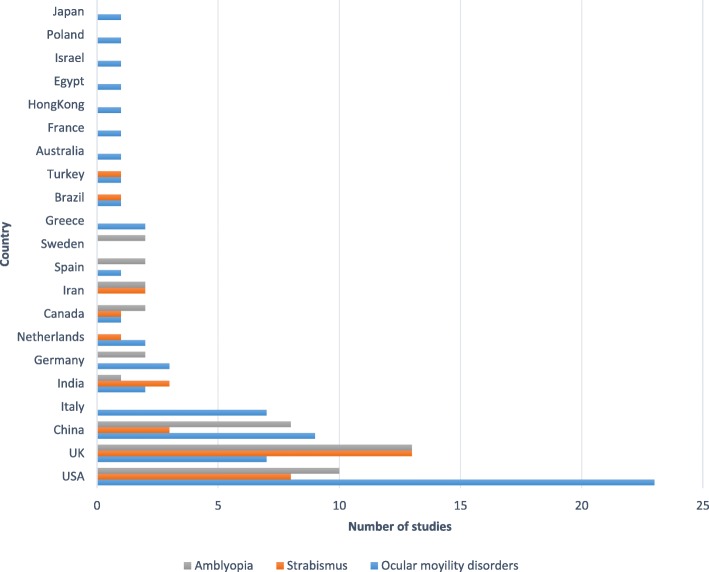
Distribution of included studies by countries where they were conducted
