# Supplementary material for: Development of a core outcome set for amblyopia, strabismus and ocular motility disorders: a review to identify outcome measures
Source: BMC Ophthalmol. 2019 Feb 8;19:47. doi: 10.1186/s12886-019-1055-8 (PMC6368710; doi:10.1186/s12886-019-1055-8)
Supplement: Supplementary file 2 — Table S2. Search terms of SCOPUS database. Terms and Boolean operators used to perform the search for the review in one example database- SCOPUS. (DOCX 19 kb) [file 12886_2019_1055_MOESM2_ESM.docx]

| **Terms** | **Boolean operator** | **Terms** |
| --- | --- | --- |
| Outcome*  Measur*  Assess*  Treat*  Interven*  Manag*  Diagnos*  Test*  Screen*  Therap*  Evaluat*  Clinic* | AND | *Amblyopia* [Mesh term]   - Strabismic - Anisometropic - Meridional - Ametropic - Stimulus deprivation   *Strabismus* [Mesh term]   - Heterophoria - Heterotropia - Esotropia/esophoria - Exotropia/exophoria - Hypertropia/hyperphoria - Hypotropia/hyperphoria - Cyclotropia/cyclophoria - Microtropia - Convergent strabismus - Divergent strabismus - Vertical strabismus   *Ocular motility disorders* [Mesh term]   - Acquired nystagmus (all types) - Horizontal gaze palsy - Internuclear ophthalmoplegia - Vertical gaze palsy - Double elevator palsy - Dorsal midbrain syndrome - Double depressor palsy - Inverse dorsal midbrain syndrome - One and a half syndrome - Third nerve palsy - Fourth nerve palsy - Sixth nerve palsy - Convergence paralysis - Convergence spasm - Convergence insufficiency - Divergence paralysis - Skew deviation - A pattern - V pattern - X pattern - Y pattern - Thyroid eye disease - Blow out fracture - Myasthenia gravis and eye - Duane’s syndrome - Brown’s syndrome - Ocular myositis |

Search terms of SCOPUS database
