# Supplementary material for: Development of a core outcome set for amblyopia, strabismus and ocular motility disorders: a review to identify outcome measures
Source: BMC Ophthalmol. 2019 Feb 8;19:47. doi: 10.1186/s12886-019-1055-8 (PMC6368710; doi:10.1186/s12886-019-1055-8)
Supplement: Supplementary file 3 — Table S3.1. Amblyopia included studies. Included studies for amblyopia arranged by type of study, study ID, title, outcome measure domain, outcome measurement and time of measurement. (DOCX 30 kb) [file 12886_2019_1055_MOESM3_ESM.docx]

| *Type of study* | *Study ID* | *Title* | *Outcome measure*  *domain* | *Outcome measurement* | *Time of measurement* |
| --- | --- | --- | --- | --- | --- |
| **Amblyopia Cochrane reviews** | Liu at al 2011  (protocol) | Acupuncture for amblyopia in children | **-BCVA**  **-Stereopsis**  **-Adverse events**  **-QoL** | -In sec of arc  -Needling pain, bleeding  -e.g SF-36 | At 6 months/ any period |
|  | Taylor et al 2012 | Interventions for unilateral and bilateral refractive amblyopia | **-BCVA**  **-Adverse events**  **-QoL**  **-Economic data** | -Age appropriate.  -From refractive or occlusion treatment | At 12 months/any period |
|  | Antonio Santos et al 2014 | Occlusion for stimulus deprivation amblyopia | **-BCVA**  **-Stereopsis**  **-Cost data**  **-Adverse effects**  **-QoL** | -Age appropriate (Log Mar)  -Any measure  -From refractive or occlusion treatment /psychological/cessation of treatment from poor compliance/diplopia/allergy to patches | At 12 months/  Any period |
|  | Taylor et al 2014 | Interventions for strabismic amblyopia | **-BCVA**  **-Adverse effects**  **-QoL**  **-Economic data** | -Age specific  Psycho/allergic/disorientation/diplopia/occlusion amblyopia  -Related to amblyopia/treatment  -Cost of treatment | At 12 months/  Any period |
|  | Korah et al 2014 | Strabismus surgery before versus after completion of amblyopia therapy in children | **-Orthotropia or microtropia**  **-BCVA**  **-Motor fusion**  **-Sensory fusion**  **-Stereopsis**  **-Adverse events** | -Cover test  -Log Mar or equivalent  -Base out or base in prism test/ synoptophore/ or other standard test  -Bagolini glasses/ Worth's 4 dot test  -Titmus fly test | At 3 years f/u |
|  | Tailor et al 2015 | Binocular versus standard occlusion or blurring treatment for unilateral amblyopia in children aged 3-8 years | **-D BCVA**  **-Stereopsis**  **-Compliance**  **-Adverse events**  **-QoL**  **-Cost effectiveness** | -Age appropriate. (Log Mar)  -In sec of arc  -Occlusion dose monitor , electronic compliance, or parent rx dia diplopia/dec VA  -Validated tool  -Of treatment | At 12 months/  Any period |
| *Type of study* | *Study ID* | *Title* | *Outcome measure domain* | *Outcome measurement* | *Time of measurement* |
| **Amblyopia Systematic reviews and meta-analysis** | Alió et al 2011 | Ped Ref Sx and its Role in the rx of Amb: Meta-analysis | **-CDVA & UCVA**  **-Refractive outcome**  **-Postoperative complications** | -(log MAR)  -Spherical and cylindrical refraction | Between 3 and 54  Months |
|  | Carlton et al 2011 a | HRQoL in patients with amblyopia and strabismus: a systematic review | **-QoL** | -(A&SQ), (ATI), (AS-20), (IXTQ) | Not given |
|  | Carlton et al 2011 b | Amblyopia and quality of life: a systematic review | **-QoL** (impact on family life, social interactions, undertaking daily activities, as well as feelings and behaviour) | -CVFQ, SPPC, VF-14, (ATI), (A&SQ), Psych Impact Ques, (PMT), (PSQ), qualitative methods | Not given |
|  | Holmes et al 2011 | Effect of Age on Response to Amblyopia Treatment in Children (A meta-analysis) | **-Visual acuity**  **-Amb & fellow eye spherical equivalent** | -ATS HOTV protocol/ E-ETDRS protocol/Snellen equivalent | Around 17-24 weeks |
|  | West et al 2011 | Amblyopia (A systematic review) | **-Visual acuity**  **-Stereopsis**  **-Compliance**  **-Adverse effects**  **-Emotional and**  **behavioural impact**  **-Social stigma scores** | -Log Mar  -e.g. Skin irritation  -Mean Rutter behaviour scores/ a questionnaire |  |
|  | Yan et al 2013 | A Meta-Analysis of Randomized Controlled Trials on  Acupuncture for Amblyopia | **-Efficacy and safety of acupuncture for amblyopia** | Not given | From 18 months to 3 years |
|  | Yang et al 2013 | Efficacy and tolerance of levodopa to treat amblyopia:  a systematic review and meta-analysis | **-Visual acuity**  **-(VEP) latency**  **-Adverse events** | -Log MAR: Snellen VA charts/ ETDRS charts  -Headache/ Sleepiness/ Nausea/ Dizziness | Endpoint (not clear) |
|  | Tsirlin et al 2015 | Behavioral Training as New Treatment for Adult  Amblyopia: A Meta-Analysis and Systematic Review | **-Visual acuity**  **-Stereo- sensitivity** | -Log MAR/Snellen Line by Line/Snellen Letter by Letter/ETDRS Letter by Letter/ETDRS Line by Line/Bailey-Lovie Letter by Letter/Tumbling E Letter by Letter  -Arc sec/octaves((an octave is a halving/doubling of the score)/Preschool Randot/Near Frisby/Far Frisby/Distance Randot |  |

| *Type of study* | *Study ID* | *Title* | *Outcome measure domain* | *Outcome measurement* | *Time of measurement* |
| --- | --- | --- | --- | --- | --- |
| **Amblyopia RCTs** | Christoff et al 2011 | Distance versus near visual acuity in amblyopia | **-BCVA**  **at distance**  **& near** | -single-surrounded HOTV optotypes (logMAR)  at 3 meters  at 0.4 meters | Prior to initiating the protocol-prescribed therapy |
|  | Evans et al 2011 | Randomised controlled trial of intermittent photic stimulation for treating amblyopia in older children and adults | **-VA (log MAR)** | -A sensitive staircase measure  of visual acuity (VA), The Crowded Acuity Test (Glasgow Acuity Test) at 3 m | Post-treatment VA  occurred 1 week after the last treatment session,  mean interval to follow-  up was 1 year 9 months (range 7 months to 3 years 3 months) |
|  | Gong et al 2011 | Observation on therapeutic effect of child amblyopia treated with auricular point sticking therapy | **-Changes of vision**  (abstract only) |  | 3 years |
|  | Lam et al 2012 | Adjunctive Effect of Acupuncture to Refractive Correction on Anisometropic Amblyopia : One-Year Results of a Randomized Crossover Trial | **-BCVA** | -LogMAR (HOTV testing protocol) | 15, 30, and 60 weeks |
|  | Medghalchi et al 2011 | A randomized Trial of Atropine versus patching for treatment of moderate amblyopia | **-Success is an increase of 2 or more lines of visual acuity or a final visual acuity of 20/25 or better**  **-Stereoacuity** | -An E chart (Nidek projector), in Log MAR Any measure  -Seconds of arc | 2 years |
|  | Wu et al 2011 | Clinical study on electrical plum-blossom needle for treatment of amblyopia in children | **-Clinical effect**  (abstract only) |  | 1-month |
|  | Bau et al 2012 | Effectivity of an Occlusion-Supporting PC-Based Visual Training Programme by Horizontal Drifting Sinus Gratings in Children with Amblyopia | **-Visual acuity**  (abstract only) |  | - |
|  | Ivandic et al 2012 | Low-Level Laser Therapy Improves Visual Acuity in Adolescent and Adult Patients with Amblyopia | **-Best corrected distant visual acuity**  **-M-VEP :multifocal visual evoked potential (amplitude and latency)** | -Snellen projection optotypes at a 20-foot distance transformed into log MAR | Data was collected during a 7-year period (maximal follow-up of 13 years) |
|  | Agervi et al 2013 | Two-year follow-up of a  randomized trial of spectacles  plus alternate-day patching to  treat strabismic amblyopia | **-Median change in VA of the amblyopic eye (distance vision)**  **VA of the amblyopic and fellow eyes**  **-Binocularity**  **-Refractive errors** | -LogMAR USING a linear Lea  symbols 15-line folding distance chart  -The Lang stereo  test II and examination with Bagolini  glasses at distance and near (seconds of arcs)  -Median SE refractive error | 2-year |
|  | Agervi et al 2013 | Two-year follow-up of a  randomized trial of spectacles alone or combined with Bangerter filters for treating anisometropic amblyopia | **-Median change in VA of the amblyopic eye (distance vision)**  **VA of the amblyopic and fellow eyes**  **-Binocularity**  **-Refractive errors** | -Log MAR USING a linear Lea  symbols 15-line folding distance chart  -The Lang stereo  test II and examination with Bagolini  glasses at distance and near (seconds of arcs)  Median SE refractive error | 2-year |
|  | Foss et al 2013 | I-BiT™, children ,amblyopia study protocol | **Uniocular assessment of visual acuity test (aided with glasses if applicable)**  **-Binocular functions**  **-Patient satisfaction**  **-Compliance**  **-Adverse events** | -Log MAR units  -Patient satisfaction questionnaire |  |
|  | Stewart et al 2013 | The effect of amblyopia treatment on stereoacuity | **-Log MAR visual acuity**  **-Stereoacuity** | -Three-letter log MAR visual acuity charts  were used: ETDRS  , crowded, and single log MAR (age-dependent).  -Frisby stereotest |  |
|  | Wallace et al 2013 | A Randomized Trial of Increasing Patching for Amblyopia | **-Best-corrected visual acuity (VA) in the amblyopic eye**  **-Ocular alignment**  **-Stereoacuity**  **-Compliance with patching treatment** | -Log MAR (without cycloplegia using the participant's optimal spectacle correction (if applicable) by isolated crowded Amblyopia Treatment Study HOTV (for subjects aged 3 to <7 years) or Electronic Early Treatment Diabetic Retinopathy Study (for subjects aged 7 to <10 years) optotypes  -Simultaneous prism and cover test at distance and near  -The Randot Preschool Stereoacuity Test (Stereo Optical Co., Chicago, IL)  Excellent (>75%), good (51%–75%), fair (26%–50%), or poor (≤25%). (parent, who recorded the numbers of hours ) | After 10 weeks. |
|  | Jafari et al 2014 | CAM visual stimulation with conventional method of occlusion treatment in amblyopia: A randomized clinical trial | **-Visual acuity improvement/**  **reduction of amblyopia severity**  **-Stereopsis improvement**  (abstract only) | -Log- MAR  -Seconds of arc |  |
|  | Mansouri et al 2014 | Binocular Training Reduces Amblyopic  Visual Acuity Impairment | **-Mean visual acuity (in Log MAR)** | -A computerized Log MAR chart (PVVAT  Precision Vision Inc) | At the end of the 6-week training period, and 6 months following the cessation  of binocular training |
|  | Pawar et al 2014 | Effectiveness of the addition of citicoline to patching in the treatment of amblyopia around visual maturity: A randomized controlled trial | **-Visual acuity** | -In log MAR | Every month in phase 1 till plateau was achieved and then for 12 months in phase 2 |
|  | Pradeep et al 2014 | An educational intervention to improve adherence to high-dosage patching regimen for amblyopia: a randomised controlled trial | **-Compliance**  **-Visual acuity** | -Electronic occlusion dose monitor (ODMs) recordings (expressed as percentage of prescribed hours patched)  -Glasgow log MAR crowded acuity cards (Keeler, Windsor, UK) | Over the 12-week period |
|  | Pediatric Eye Disease Investigator Group 2014 | Atropine vs Patching for Treatment of Moderate Amblyopia  Follow-up at 15 Years of Age of a Randomized Clinical Trial | **-Mean Visual acuity at 15 years of age**  **-Manifest and cycloplegic refractions**  **-Ocular alignment**  **-Stereoacuity** | -Log MAR lines, the electronic ETDRS  -If visual acuity in either eye  Was worse than 20/20(<83 letters) or if an IOD between the eyes of 5 or more letters was found, a manifest refraction was performed that included dry retinoscopy with subjective refinement  -The simultaneous prism and  cover test at distance and near fixation  T-he Randot Preschool Stereoacuity Test (Stereo Optical Company). | At the 15-  year examination |
|  | Han et al 2015 | Randomized Controlled Clinical Trials for Treatment of Child Amblyopia with Otopoint Pellet-pressure Combined with Chinese Medical Herbs | **-Clinical effect**  **-Improvement degree of vision**  **-Onset time of improvement**  **-Recurrence rate**  (abstract only) |  | In the 3 and 6 months' follow-up visit |
|  | Moseley et al 2015 | Personalized versus standardized dosing strategies for the treatment of childhood amblyopia: study protocol for a randomized controlled trial | **-Visual acuity** | -Assessed on one or more of the following test charts: crowded log MAR, uncrowded log MAR, or modified ETDRS. | Participants will have their visual acuity recorded every 2 weeks until the trial endpoint is reached |
|  | PEDIG 2015 a | A Randomized Trial of Levodopa as Treatment for Residual Amblyopia in Older Children | **-Mean change in best-corrected amblyopic-eye VA**  **-Ocular alignment**  **-Stereoacuity**  **-Adverse events** | -The Electronic Early Treatment of Diabetic Retinopathy Study VA protocol  -The simultaneous prism and cover test  -The Randot Preschool Stereotest (Stereo Optical Co, Inc, Chicago, IL)  -A symptom survey was completed by the participant and by the parent ,17 items with a 5-level Likert scale ,an average score was calculated | At 18-week, follow-up continued through 26 weeks |
|  | PEDIG 2015 b | A randomized trial of adding a plano lens to atropine for amblyopia | **-Amblyopic-eye visual acuity**  **-Ocular alignment**    **-Stereoacuity**  **-Compliance**  **-Adverse events** | -Using the participant’s best spectacle correction by a study-certified tester using the ATS-HOTV11 protocol on the Electronic Visual Acuity Tester  -Measured at distance and near with a simultaneous prism and cover test  -The Randot Preschool Stereoacuity Test (Stereo Optical Co, Chicago, IL)  -Based on discussions with the parent and by reviewing study calendars maintained by the parent, was judged to be excellent (>75%), good (51%-75%), fair (26%-50%), or poor (≤25%) | At 10 weeks  At 12 weeks |
|  | Herbison et al 2016 | Randomised controlled trial of video clips and interactive games to improve vision in children with amblyopia using the I-BiT system | **-The difference in visual acuity improvement between the three arms**  **-Change in VA**  **-Double vision**  **-Drop in vision** | -Log MAR | At week 6 |
|  | Tang et al 2016 | A comparative study on visual acuity and stereopsis outcomes between perceptual learning based on cloud services and conventional therapy for amblyopia | **-Visual acuity improvement**  **-Stereopsis**  (abstract only) |  |  |

| *Type of study* | *Study ID* | *Title* | *Outcome measure domain* | *Outcome measurement* | *Time of measurement* |
| --- | --- | --- | --- | --- | --- |
| **Amblyopia non-systematic reviews** | C. E. Stewart et al 2011 | Amblyopia Therapy: An Update | **-Best visual acuity**  **-Visual outcomes**  **-Health-related quality of life (HRQol) measures**  **-Compliance/ Concordance** | -Log MAR  -Amblyopia Treatment Index (ATI) parental questionnaire  -Objective occlusion dose monitoring |  |
|  | Matta et al 2013 | Part- time vs. Full- time  Occlusion for Amblyopia:  Evidence for Part- time Patching | **-Lines improved in VA** |  |  |
|  | DeSantis et al 2014 | Amblyopia (summary of PEDIG studies) | **-Visual acuity (in amblyopic eye OR Binocular VA)**  **-Sensory outcomes** | -Snellen eye chart  Allen figures  Tumbling E charts  HOTV charts | At 2 years |
|  | Sanchez et al 2016 | Advantages, limitations, and diagnostic accuracy of photoscreeners in early detection of amblyopia: a review | **-Detection of amblyopia**  **(with a simple outcome report: retest, pass, or refer)** | MTI photoscreener  PowerRefractor (Plusoptix, Nuremberg, Germany)  PowerRefractor II (Plusoptix)  Plusoptix photoscreener series  iScreen  Spot  2WIN | N/A |
