# Supplementary material for: Development of a core outcome set for amblyopia, strabismus and ocular motility disorders: a review to identify outcome measures
Source: BMC Ophthalmol. 2019 Feb 8;19:47. doi: 10.1186/s12886-019-1055-8 (PMC6368710; doi:10.1186/s12886-019-1055-8)
Supplement: Supplementary file 4 — Table S3.2. Strabismus included studies. Included studies for strabismus arranged by type of study, study ID, title, outcome measure domain, outcome measurement and time of measurement. (DOCX 34 kb) [file 12886_2019_1055_MOESM4_ESM.docx]

| *Type of study* | *Study ID* | *Title* | *Outcome measure domain* | *Outcome measurement* | *Time of measurement* |
| --- | --- | --- | --- | --- | --- |
| **Strabismus Cochrane reviews** | Rowe et al 2012 | Botulinum toxin for the treatment of strabismus | **-Ocular alignment**  **-BSV**  **-QoL**  **-Economic data**  **-Adverse events** | -Prisms or synoptophore  -Cover test, motor fusional vergences and stereoacuity  -Any measure  -Ptosis, induced vertical deviation, subconjunctival haemorrhage, intolerable diplopia | Minimum 6 months |
|  | Elliot et al 2013 | Interventions for infantile esotropia | **-Ocular alignment**  **-Binocularity**  **-No. of interventions required**  **-QoL**  **-Adverse events** | -Prism cover test, prism reflections or synoptophore  -1) stereoacuity (gold standard), 2) motor fusion 3)simultaneous perception  -(sx: ant.segment ischemia, conjunctival scarring, inflammation), (Botox: ptosis), development of amblyopia | At 6 months |
|  | Haridas et al 2013 | Adjustable versus nonadjustable sutures for strabismus | **-Ocular alignment**  **-Re-operation rates**  **-Post op complications**  **-Economics**  **-Patient satisfaction** | -Any method  -Diplopia, hmrg, scleral perforation, ant segment ischemia, lost muscles, subconj cysts, stitch reactions  -Any validated measurement scale | At 6 months |
|  | Hatt et al 2013 | Interventions for intermittent exotropia | **-Alignment at near and distance**  **-Stereoacuity at near & distance**  **-Motor fusion test at near or distance or both**  **-Adverse events**  **-QoL** | -Simultaneous or alternate cover test or both or using synoptophore  -Any age appropriate test  -Overaction/amblyopia/intra-op complications.  -Any measure | Any period |
|  | Jones-Jordan et al 2014 | Spectacle correction versus no spectacles for prevention of strabismus in hyperopic children | **-Manifest strabismus**  **-Amblyopia (VA)**  **-Stereoacuity**  **-QoL** | -Unilateral cover test or the Hirschberg test in conjunction with the 4-D prism test for suppression or microtropia  -Difference as 3 lines of VA b/w eyes  -Randot stereoacuity test or a similar measure | After treatment period of min of 3 yrs. |
|  | Korah et al 2014 | Strabismus surgery before versus after completion of amblyopia therapy in children | **-Orthotropia or microtropia**  **-BCVA**  **-Motor fusion**  **-Sensory fusion**  **-Stereopsis**  **-Adverse events** | -Cover test  -Log Mar or equivalent  -Base out or base in prism test/ synoptophore/ or other standard test  -Bagolini glasses/ Worth's 4 dot test  -Titmus fly test | At 3 years f/u |
|  | Tailor et al 2014  (protocol) | Tests for detecting strabismus in children age 1 to 6 years in the community | **-Ocular misalignment**  **-Stereopsis**  **-VA tests**  **-Asymmetry of corneal reflections** | -Hirschberg test/Cover test/Cover-uncover test/Simultaneous prism and cover test  -Contour tests  -In Log MAR or log MAR equivalent  -Automated refraction devices |  |
|  | Hatt et al  2015 | Interventions for dissociated vertical deviation | **-Decreased hyperdeviation to 4pd or less**  **-Binocularity**  **-Proportion of day of misalignment**  **-QoL**  **-Adverse events** | -Prism under cover test/prism and alternate cover test  -Any binocular vision test  -Vision-specific QoL instruments: NEI, VFQ25, AS-20, and instruments specific for children | One year from intervention |
|  | MacKenzie et al 2016 | Psychological interventions for improving quality of life outcomes in adults undergoing strabismus surgery | **-QoL**  **-Anxiety**  **-Depression**  **-Social anxiety and social avoidance**  **-Success in terms of desired surgical outcome** | -Self-admin QoL questionnaires, for e.g. (VFQ-25), (NEi-VFQ), (VF 14)/ AS-20, (A&SQ), (SF), (EQ5D)  -10 PD of emmetropia and absence of diplopia in PP and in down gaze | 3-6 months following surgery and 1 year or more where available |

| *Type of study* | *Study ID* | *Title* | *Outcome measure domain* | *Outcome measurement* | *Time of measurement* |
| --- | --- | --- | --- | --- | --- |
| **Strabismus systematic reviews** | Tadić V et al 2013 | PROMs in paediatric ophthalmology: a systematic review | **-Patient-reported and parent proxy-reported outcome measures**  **-Measures of impact of living with visual impairment or an ophthalmic condition including quality of life (QoL)** | -Impact of Vision Impairment on Children (IVI_C)  Children’s Visual Function Questionnaire (CVFQ),Intermittent Exotropia Questionnaire (IXTQ),  Pediatric Refractive Error Profile (PREP), Children’s Amblyopia Treatment Quality of Life Questionnaire (CAT-QoL), Perceived Psychosocial Questionnaire (PPQ),Cardiff Visual Ability Questionnaire for Children (CVAQC) LV Prasad–Functional Vision Questionnaire (LVP-FVQ),  LV Prasad–Functional Vision  Questionnaire Second Version (LVP-FVQ II),  Effects of Youngsters’ Eyesight on Quality of Life (EYE-Q), Amblyopia Treatment Index (ATI), Emotional Impact of Amblyopia Questionnaire (EIAQ),Psychological Impact Questionnaire (PIQ) |  |
|  | Chiu et al 2014 | Standardising reported outcomes of surgery for intermittent exotropia | **-Alignment**  **-Near stereoacuity**  **-Control score**  **-Quality of life score and patient-reported outcome measure (IXTQ score)** | -Alternate prism cover test for near and distance  -Office Control Score/ Newcastle Control Score  -IXTQ score | Ranged from two months to two years after  surgery |
|  | McBain et al 2014 | The impact of strabismus on quality of life in adults  with and without diplopia: a systematic review | **-Quality of life (QoL)** | -SF-36, SF-8, SF-12, EUROQoL EQ-5D, Visual Function Questionnaire (VFQ-25), A shortened version of the National Eye Institute Visual FunctionQuestionnaire30  (NEI-VFQ), the 14-item Visual Function questionnaire (VF-14),  Four strabismus-and amblyopia-specific QoL questionnaires: the amblyopia and strabismus questionnaire (A&SQ), the amblyopia treatment index, the adult strabismus questionnaire (AS-20), and the intermittent exotropia questionnaire |  |
|  | Joyce KE et al 2015 | A systematic review of the effectiveness  of treatments in altering the natural history  of intermittent exotropia | **-Angle of deviation**  **-Stereoacuity**  **-Control**  **-Adverse effects** |  | At least 6 months follow up |

| *Type of study* | *Study ID* | *Title* | *Outcome measure domain* | *Outcome measurement* | *Time of measurement* |
| --- | --- | --- | --- | --- | --- |
| **Strabismus**  **RCTs** | Nabie et al 2011 | Anchored versus conventional hang-back bilateral lateral rectus muscle recession for exotropia | **-Horizontal and vertical deviation**  **-Induced A or V pattern**  **-Complications**  **-The overall success rate** | -For distance (6 m) and near (1/3 m) with prism and alternate cover testing  -Such as globe perforation and vertical deviations  -Deviation within 10 D of orthotropia | At 1.5, 3, and 6 months |
|  | Rajavi et al 2011 | A Randomized Clinical Trial Comparing Myectomy and Recession in the Management of Inferior Oblique Muscle Overaction | **-IO action**  **-Horizontal or vertical deviations**  **-V pattern**  **-DVD**  **-Stereopsis** | -An ordinal scale from 0 to 4+. Final IO function with grade 0 or 1+ was defined as a satisfactory result  -For measuring eye deviation at primary position, prism alternate cover or Krimsky tests were used depending on whether BCVA was 20/200 or better or worse than 20/200, respectively  -Titmus test | At month 3 |
|  | Saxena et al 2011 | Evaluation of factors influencing distance stereoacuity  on Frisby-Davis Distance Test (FD2) in intermittent exotropia | **-Deviation was measured for near and distance**  **-Near stereoacuity**  **-Distance stereoacuity**  **-Fusional vergence** for both convergence and divergence was measured at distance (6 m) and near fixation (33 cm) | -Prism Bar Cover Test for both near (33 cm) and distance (6 m). The PBCT was done with spectacle correction in patients who were using glasses. A patch test (occlusion for 6 h) was used whenever there was any disparity between the near and distance deviation to rule out pseudodivergence excess, while measurement was repeated with the addition of +3 dioptre in cases with a high AC/A ratio.  -The Netherland Organization stereo test (TNO) s of arc (with subjects wearing red-green spectacles)  -The Frisby-Davis Distance (FD2) stereo test (s of arc) (Readings were also recorded monocularly to rule out the possibility of a subject using monocular cues)  -With a prism bar | At 1 week, 1 month, 3 months and 6 months. |
|  | Buck et al 2012 | Surgery versus Active Monitoring in Intermittent Exotropia (SamExo): study protocol for a pilot randomised controlled trial | **-Ocular misalignment**  **-Control**  **-Age-specific QOL assessments**  **-Rates of amblyopia**  **-Cost**  **-Adverse events** | -The APCT  -The NCS and Mayo scores, parental report  -NHS costs, use of health-care resources, costs to families  -Perforation of the globe occurring within 24 h, intraocular infection occurring within 2 weeks, lost or slipped muscle Occurring within 1 month, scleritis occurring within 1 month, becoming constant XT occurring within 9 months, persistent over-correction occurring within 9 months, re-operation for under or over-correction occurring within 9 months | 3-, 6- and 9-month |
|  | Minguini N et al 2012 | Surgery with intraoperative botulinum toxin-A  injection for the treatment of large-angle horizontal  strabismus: a pilot study | **-Angles of deviation (measured with the best optical correction in place for the distance and the near and cardinal gaze**  **-Visual acuity, (Fixating and non-fixating eye BCVA)**  **-Adverse effects** | -The simultaneous and alternate-cover tests  -The Krimsky test was employed when the cover tests were not applicable.  -Snellen visual acuity was converted to LogMAR acuity prior to statistical analysis and then converted back to the Snellen equivalent  -Ptosis | One day, two weeks, one month, three  months, and 6-12 months |
|  | Chen et al 2013 | Botulinum toxin injections combined with or without sodium hyaluronate in the absence of electromyography for the treatment of infantile esotropia: a pilot study | **-Size of deviation**  **-Ocular movements**  **-Complicated ptosis**  **-Induced vertical deviation** | -Prism and alternate cover tests or the Krimsky or Hirschberg estimate | At 2 weeks, 3 months, and 6 months |
|  | PEDIG  2014 | A randomized trial comparing part-time patching with observation for children 3 to 10 years of age with intermittent exotropia | **-Stereoacuity**  **-Exotropia control** (at distance and near)  **-Ocular alignment** | -Distance stereoacuity : the Distance Randot test at 3 meters  Near stereoacuity was assessed using the Preschool Randot test, Titmus Fly, and Titmus Circles tests at 40 centimetres.  -Office Control Score  -Cover/uncover test, simultaneous prism and cover test (SPCT), and PACT | 6 months (unless there was deterioration before) |
|  | Wang et al 2014 | Comparison of different surgery procedures for convergence insufficiency-type intermittent exotropia in children | **-Angle of deviation**  **-Near stereopsis**  **-Lateral incomitance**  **-Complications** | -The prism and alternate cover test (PACT )at both distance and near ,PACT was performed again after 1 h monocular occlusion of the non-dominant eye  -Randot stereotest | 6 months |
|  | Clarke et al 2015 | An external pilot study to test the feasibility of a randomised controlled trial comparing eye muscle surgery against active monitoring for childhood intermittent exotropia [X(T) | **-Stereoacuity**  **-Ocular alignment**  **-Control**  **-Age-specific QoL assessments**  **-Patient-derived outcomes**  **-Rates of amblyopia**  **-Economic outcomes**  **-Adverse events** | -In PDs for near and distance fixation, by a simultaneous prism cover test, which attempts to capture the alignment before binocular vision is disrupted, or on an alternating prism cover test, which is the total misalignment demonstrated by disrupting binocular vision by covering each eye in turn. The usual reported measure is the total misalignment on an alternating prism cover test  -NCS and the Mayo Score and by parental report  -The Intermittent Exotropia Questionnaire  -e.g. acceptability and adherence  -Use of health-care resources, NHS costs, costs to families accessing the treatments being evaluated and incremental cost per cured patient and a cost–consequences analysis  -In accordance with the National Research Ethics Service guidelines |  |
|  | Gross et al 2015 | Induced Incomitance of one Muscle Strabismus Surgery in Comparison to Unilateral Recess-Resect Procedures | **-Induced incomitance**  (abstract only) | -The latent angle of squint on a tangent screen over the horizontal 60° in 10° increments and then calculated the amount of induced incomitance | 3 months |
|  | Yilmaz et al 2015 | The impact of prism adaptation test on surgical outcomes in patients with primary exotropia | **-Surgical success rates**  **-Angle of deviation**  **-Binocular single vision /Stereopsis** | -Deﬁned as ocular alignment within eight PD of orthophoria at distance  -The prism and alternate cover test  -TNO test (arc sec)/Fusion by Bagolini’s striated glass | Six months and One year  At one year postoperatively |
|  | Mohney et al 2015 | A Randomized Trial Comparing Part-time Patching with Observation for Intermittent Exotropia in Children 12 to 35 Months of Age | -**Deterioration** | -Defined as constant exotropia measuring at least 10 Δ at distance and near or receipt of non-protocol treatment for IXT by SPCT, confirmed by a retest | At either the 3- or 6-month visit |
|  | Wang et al 2015 | Effectiveness of strabismus surgery on the health-related quality of life assessment of children with intermittent exotropia and their parents: a randomized clinical trial | **-Change in (intermittent exotropia questionnaires) IXTQ score** | -IXTQ (accessible via www.pedig.net) | 3 months |

| *Type of study* | *Study ID* | *Title* | *Outcome measure domain* | *Outcome measurement* | *Time of measurement* |
| --- | --- | --- | --- | --- | --- |
| **Strabismus non-systematic reviews** | Piano et al 2011 | Conservative Management  of Intermittent Distance  Exotropia: A Review | **-Control scores to quantify the severity of IDEX**  **-Angle of deviation/motor outcomes**  **-Binocularity/sensory outcomes**  **-AC / A ratios**  **-VA**  **-Appearance of the**  **Strabismus**  **-Suppression**  **-Fusion**  **-Convergence**  **-Positive fusion amplitudes**  **-Accommodative/ fusion facility**  **-Positive relative**  **Convergence**  **-Control of the near angle** | -Petrunak and Rao’s five point scale, the Holmes and Mohney Office Control Scale, and the Newcastle Control Score.  -TNO stereoacuity  -Log MAR |  |
|  | Simonsz et al 2011 | Best age for surgery for infantile esotropia | **-Binocular vision**  -**Postoperative angle** **of strabismus**  -**Long term stability of the angle**  -**Number of operations** needed to reach these goals  or the chance of spontaneous reduction of the strabismus into a microstrabismus without surgery | -Titmus Housefly | Age of 6 years |
|  | Christoff et al 2014 | DVD—a conceptual, clinical, and surgical overview | **-Measurement of the**  **angle of deviation**  **-Control of DVD**  **-Latent nystagmus**  **-Visual acuity**  **-Motility examination**  **-Sensory status**  **-Abnormal head posture**  **-Cosmetic insecurities**  **-Discomfort/abnormal**  **sensory experiences** | -A true prism and alternate cover test (PACT)/an adapted prism and alternate cover test with each eye fixing  The estimation technique, also helps determine the control of DVD, grading scale of 1-4  DHD is measured by reversed fixation test (RFT)  -Whether the deviation is latent/manifest  -Video-oculography |  |
|  | Gunton et al 2014 | Impact of strabismus surgery on health-related  quality of life in adults | **-Health-related quality of life** | -The Amblyopia and Strabismus Questionnaire  The Adult Strabismus-20 | 1 year postoperatively |
|  | Shainberg et al 2014 | Nonsurgical Treatment of  Teenagers with High AC / A  Ratio Esotropia | **-Visual and binocularity outcomes**  **-Quality of life** | -Quality of life surveys |  |
|  | Hug  2015 | Management of infantile esotropia | **-Development of stereopsis**  **-Surgical success**  **-Angle of deviation**  **-Complication** | -Ptosis, vertical deviation, diplopia, and  subconjunctival haemorrhage |  |
|  | Kelkar et al 2015 | Intermittent exotropia: Surgical treatment strategies | **-Ocular alignment**  **-Binocularity/static distance stereoacuity/(at near or distance)/sensory outcome**  **-Fusional control/fusion amplitudes**  **-Recurrence of exotropia** | -The Newcastle Control Score/size of the deviation, duration of manifest deviation and ease of regaining fusion after dissociation from the cover‑uncover test |  |
