# Supplementary material for: Development of a core outcome set for amblyopia, strabismus and ocular motility disorders: a review to identify outcome measures
Source: BMC Ophthalmol. 2019 Feb 8;19:47. doi: 10.1186/s12886-019-1055-8 (PMC6368710; doi:10.1186/s12886-019-1055-8)
Supplement: Supplementary file 5 — Table S3.3. Ocular motility disorders included studies. Included studies for ocular motility disorders arranged by sub-condition, study ID, title, outcome measure domain, outcome measurement and time of measurement. (DOCX 50 kb) [file 12886_2019_1055_MOESM5_ESM.docx]

| *Sub-*  *condition* | *Study ID* | *Title* | *Outcome measure domain* | *Outcome measurement* | *Time of measurement* |
| --- | --- | --- | --- | --- | --- |
| Accommodation & convergence disorders  7 | Scheiman et al 2011 | Non- Surgical Interventions for Convergence Insufficiency | **-Near point of convergence**  **-Positive fusional vergence at near**  **-Patient symptoms**  **-Compliance to treatment**  **-QoL**  **-Adverse events** | -(CISS) Version-15  Diplopia/headaches/convergence spasm | At 12 weeks of intervention |
|  | Scheiman et al 2011 | Treatment of Accommodative Dysfunction in Children: Results from a Randomized Clinical Trial | **-Amplitude of accommodation**  **-Accommodative facility** | -By the push-up method using a moveable target of 20/30 letters on the Astron Accommodative Rule (Gulden Ophthalmics, Elkins Park, PA) Decreased accommodative amplitude was defined as 2.00 D below the lowest expected amplitude based on the Hofstetter’s formula of 15 to 1/4 age  -Accommodative facility was the speed at which the patient could see 20/30  letters at 40 cm clearly through alternating 2.00 D and 2.00 D lenses, measured in cycles per minute (cpm) (i.e., of 2.00 D and  2.00 D). Decreased accommodative facility was defined as 6 cpm, which is 1 SD below the normative value of 11 cpm for school-age children | After 4, 8, and 12 weeks of treatment. And One year after completion of therapy |
|  | Barnhardt et al 2012 | Symptoms in Children with Convergence Insufficiency: Before and After Treatment | **-Frequency and severity of symptoms**  **-Mean change in performance-related vs. eye-related symptoms for treatment responders** | -The convergence insufficiency symptom survey (CISS) | At baseline and post treatment. At one year follow up. |
|  | Cooper et al  2012 | Convergence insufficiency--a major review | **-**Phoria (exophoria that is greater at near than distance)  **-Near point of convergence** (a remote near point of convergence (NPC), i.e., a breakdown in convergence greater than 3 inches)  **-Fusional convergence and Fusional recovery** (decreased positive fusional convergence (PFC) at near)  **-** **CI Symptom Survey score**  **-Near point analysis**  **-** **Accommodation**  **-** **Sensory fusion** (CI have normal stereopsis (40  seconds of arc or better)  **-** **Refractive error** (There is no correlation between refractive error and CI)  **- Relationship to learning/attention** | Δ  **-(CISS questionnaire)**  - Contour and random dot  stereograms | 6 weeks/12 weeks |
|  | Cacho-Martíneza et al 2014 | Is there any evidence for the validity of diagnostic criteria used for accommodative and nonstrabismic binocular dysfunctions? | **-Symptoms**  **-Phoria**  **-NFV/PFV negative/positive fusional vergence**  **-NPC near point of convergence**  **-AC/A**  **-BAF binocular accommodative facility**  **-Monocular accommodative facility (MAF)**  **-MEM dynamic retinoscopy**  **-PRA/NRA: positive/negative relative accommodation**  **-Visual acuity**  **-Refractive error**  **-Low amplitude of accommodation (AA)** | -CISS V-15/ reported without questionnaires in some conditions/ no validated questionnaires for some anomalies  -Exophoria at near≥4 greater than at distance Von Graefe method |  |
|  | Horan et al  2015 | Is The Convergence Insufficiency Symptom Survey Specific for Convergence Insufficiency? A Prospective, Randomized Study | **-CISS** (Note: This study suggests that the CISS questionnaire is not specific for convergence insufficiency)  **-BCVA**  **-** **Stereopsis**  **- Manifest strabismus**  **- Convergence amplitudes at 1/3 m and 3 m**  **-NPC**  **-Accommodation amplitude**  **-** **Positive fusional vergence** | **-Titmus test**  **-cover testing at distance**  **and near,** |  |
|  | PEDIG  2016 | Home-Based Therapy for Symptomatic Convergence Insufficiency in Children: A Randomized Clinical Trial | **-CISS score**  **-Mean NPC break**  **-Positive fusional vergence at near (PFV)** |  | At 12 weeks |

| *Sub-*  *Condition* | *Study ID* | *Title* | *Outcome measure domain* | *Outcome measurement* | *Time of measurement* |
| --- | --- | --- | --- | --- | --- |
| Mechanical restriction  6 | Joshi et al  2011 | Overview of Pediatric Orbital Fractures | **-**Soft tissue entrapment/ fracture  **-**Globe integrity  **-**Orbital dystopia  **-**Oculocardiac reflex  **-Visual acuity**  **-**Pupillary function  **-Extraocular motility**  **-Diplopia within 30 degrees of the primary position**  **-**Canthal placement  **-**Decreased sensation on the ipsilateral side to the upper cheek, upper palate, and upper lip  **-Post op complications** | -CT (helical CT remains as the ideal imaging technique)  -Horizontal or vertical diplopia,  restricted eye movements, nausea, vomiting, and severe pain with eye movements, FDT  -Orbital hematoma, blindness, infection of hardware, diplopia, pyogenic granuloma, ectropion, and entropion. |  |
|  | Manley et al  2011 | Brown’s syndrome | -**Motility assessment**  **-**C**linical assessment**  **-**Histologic examination | -Forced duction test (positive)  -Three-step test (negative)  -Cover/uncover tests for latent strabismus  -Monocular (ductions) and binocular (versions)  -A and V patterns  -Downshoot  -Widening of the palpebral fissure on adduction  -Compensatory head posture  -Tendon for histologic examination |  |
|  | Stotland et al 2011 | Pediatric Orbital Fractures | **-**Bony contour and alignment  -Orbital volume  - Position of the  globe  **-Herniated/entrapped orbital soft tissue** | - Forced duction testing |  |
|  | Cheung et al  2013 | A Systematic Review of the Endoscopic Management  of Orbital Floor Fractures | **-The resolution of diplopia**  **-The resolution of enophthalmos**  **-Postoperative complications** | -Clinical symptoms, *A field diplopia test* or ophthalmologic consultation  -Assessed clinically or with a Hertel or Naugle exophthalmometer  -Including blindness, paresthesias, sinusitis, infection, conversion to external approach, and need for revision surgery | The mean (range) follow-up was 7.6 months (1 week-5 years),Diplopia was assessed at 6 weeks to 3 months after the operation |
|  | Dubois et al  2015 | Controversies in orbital  Reconstruction—II. Timing of post-traumatic orbital  reconstruction: A systematic  review | **-Functional impairment (vision, extraocular muscle motility disorders, and diplopia)**  **-Cosmetic disturbance (enophthalmos)**  **-Infraorbital hypaesthesia**  **-Adverse effects** | -Such as persistent diplopia or enophthalmos |  |
|  | Wan et al  2015 | The Role of Computer-Assisted Technology in Post-Traumatic Orbital Reconstruction: A PRISMA driven Systematic Review | **-Diplopia**  **-Enophthalmos**  **-Procedure-related complications** | -Subjective diplopia  -Exophthalmometer, in millimetre  -Examples: reduced vision, suspected retrobulbar hematoma | A mean follow-up of 13 months |

| *Sub-condition* | *Study ID* | *Title* | *Outcome measure domain* | *Outcome measurement* | *Time of measurement* |
| --- | --- | --- | --- | --- | --- |
| **OMDs**  **Thyroid eye disease and orbital inflammation**    **25** | Boboridis et al 2011 | Surgical orbital decompression for thyroid eye disease | **-Success of treatment**  **-No. of post-treatment rehabilitative sx pro.**  **-Disease severity**  **-Exophthalmometry**  **-Adverse events** | -Composite outcome scores | 1 -6 months |
|  | Marcocci et al 2011 | Selenium and the Course of Mild Graves’ Orbitopathy | **-QoL**  **-Clinical Activity Score CAS**  **-Eye evaluation** | -Mean scores on the GO-QOL questionnaire (score of Visual functioning as a consequence  of diplopia, decreased visual acuity, or both/Appearance)  -CAS  -Eyelid aperture/Soft-tissue signs/Proptosis/Eye-muscle motility | 6 and 12 months |
|  | Rajendram et al 2012 | Orbital radiotherapy for adult thyroid eye disease | -Composite outcome score  **-Number of post treatment rehabilitative surgical procedures**  -Disease Severity Score  -Disease Activity Score  **-QoL**  **-Adverse events** | -e.g. squint operations for double vision  -like NOSPECS  -like CAS  -Validated questionnaires  Ocular or non-ocular | Min 3 months- max 2 yrs. |
|  | Barczynski et al 2012 | Randomized clinical trial of bilateral subtotal thyroidectomy  versus total thyroidectomy for Graves’ disease with a 5-year follow-up | -Prevalence of recurrent hyperthyroidism  **-Changes in Graves’ ophthalmopathy**  **-Adverse events** | -NOSPECS (no signs or symptoms, only signs, soft tissue involvement, proptosis,  *extraocular muscle involvement*, corneal involvement, sight loss) classification and total eye score (TES), the clinical activity score (CAS) was used to evaluate the activity of the ophthalmopathy; the CAS includes seven items (eyelid oedema, eyelid erythema, conjunctival redness, chemosis, oedema of the caruncle, spontaneous ocular pain, and pain with ocular movements  -Postoperative transient and permanent paresis of the recurrent laryngeal nerve, and postoperative hypocalcaemia and hypoparathyroidism | At 1, 3, 6, 9 and  12 months after surgery, and then annually for 5 years |
|  | Bartalena et al 2012 | Efficacy and Safety of Three Different Cumulative Doses of Intravenous Methylprednisolone for Moderate to Severe and Active Graves’ Orbitopathy | **-Efficacy**  **-Safety**  **-Changes in subjective diplopia**  **-The Clinical Activity Score** | -Objective measurement of ocular motility (ductions measured in degrees particularly elevation and abduction) and patient’s GO  QoL questionnaire  -Adverse events  -The Bahn’s and Gorman’s diplopia score | At 12 wk, then exploratory 24-wk visit |
|  | Leo et al  2012 | Outcome of Graves’ Orbitopathy after Total Thyroid Ablation and Glucocorticoid Treatment: Follow-Up of a Randomized Clinical Trial | **-GO outcome**  **-Time to GO best possible outcome and to GO improvement**  **-Additional treatments**  **-**TRAb disappearance  **-Quality of life** | -European Group On Graves Ophthalmopathy questionnaire | 88.0 -/+ 17.7 months |
|  | Fraser et al  2013 | Ocular Myositis | **-Dramatic and rapid clinical response to steroids**  **-Recurrence or chronicity, requiring repeated courses of steroids**  **-Development of fibrosis and late cicatricial sequelae** |  |  |
|  | Minakaran et al 2013 | Rituximab for thyroid-associated ophthalmopathy | -Improvement in clinical activity score  -Improvement in NOSPECS TAO score  **-Improvement in extraocular motility**  **-QoL**  **-Adverse events** | -CAS  -In degrees  -Failure rate/ decrease in CAS score | At 4, 6, 12 months |
|  | Shi et al  2013 | Effectiveness of somatostatin analogs versus placebo for graves' ophthalmopathy: A meta-analysis  (abstract only) | **-Clinical activity**  **-Proptosis**  **-Diplopia**  **-Orbital volume**  **-Intraocular pressure**  **-Visual acuity** | -Clinical activity score (CAS)  -mm |  |
|  | Shiber et al  2014 | Glucocorticoid regimens for prevention of Graves' ophthalmopathy progression following radioiodine treatment: systematic review and meta-analysis | **-New or worsening GO**  -Resolution of hyperthyroidism  -Steroid-related adverse events | -Clinical activity score (CAS) before treatment | 6-18 months |
|  | Gao et al  2014 | Meta-analysis of methylprednisolone pulse therapy for Graves’ ophthalmopathy | **-Improvement of clinical parameters**  **-The clinical activity score (CAS)** | -e.g. decrease in proptosis and  eyelid retraction of 2 mm or more, decrease in ocular pressure in up gaze, improvement in grade of soft tissue swelling, disappearance of ***diplopia in primary gaze* and/*or amelioration of eye movement and visual acuity*** |  |
|  | Wickwar et al 2014 | What Are the Psychosocial Outcomes of Treatment for Thyroid Eye Disease? A Systematic Review | **-Quality of life**  **-Long-term quality of life outcomes (up to 11 years)** | -SF-36TM, Sickness Index Profile (SIP), EQ-5D, visual analogue scale, various versions of the GO-QOL, visual-related quality of life,  appearance-related quality of life | 12 months (3-48 months) |
|  | Chen et al  2014 | Changes in Graves’ Ophthalmopathy after Radioiodine and Anti-Thyroid Drug Treatment of Graves’ Disease from 2 Prospective, Randomized, Open-Label, Blinded End Point Studies | **-Severity and activity of GO**  **-Euthyroidism, hyperthyroidism, hypothyroidism, relapse, and changes in GO** |  | During a 9–12-year follow-up |
|  | Moleti et al  2014 | Radioiodine ablation of postsurgical thyroid remnants after treatment with recombinant human TSH (rhTSH) in patients with moderate-to-severe graves' orbitopathy (GO): A prospective, randomized, single-blind clinical trial | **- Overall GO outcome**  **-Short-term GO outcomes**  **-Effects of acute rhTSH and 131I administration on GO.** | 1) Lid width 2) evaluation of soft-tissue involvement, using a comparative photographic colour atlas (www.eugogo.eu) 3) measurements of proptosis by Hertel exophthalmometer; 4) assessment of extraocular muscle function and diplopia (Gorman’s);5) evaluation of corneal involvement;6) evaluation of optic nerve involvement; and 7) eye disease activity evaluation, using the CAS | 2 months after Tx/radioiodine ablation  At 45 d, and at 3 and 6 months |
|  | Suhler et al  2014 | Rituximab Therapy for Refractory Orbital Inflammation  : Results of a Phase 1/2, Dose-Ranging, Randomized Clinical Trial | **-Disease activity**  **-Corticosteroid dose reduction by at least 50%**  **-Visual acuity**  **-Reduction in pain**  **-Participant- and physician-reported global health assessment** | -A validated orbital disease grading scale (modified Werner grading scale)  - Snellen eye charts, (improvement in Snellen visual acuity of 2 lines or more)  -A visual analogue scale (VAS)  -A visual analogue scale (VAS) | 24-week ( at 24 and 48 weeks) |
|  | Zhu et al  2014 | A prospective, randomized trial of intravenous glucocorticoids therapy with different protocols for patients with graves' ophthalmopathy | **-The response rate**  **-Clinical Activity Score [CAS]**  **-Diplopia**  **-Visual acuity** | -The modified EUGOGO patient form | Baseline, 4th and 12th week |
|  | Boboridis et al 2015 | Critical Appraisal on Orbital Decompression  for Thyroid Eye Disease: A Systematic Review  and Literature Search | **-Success rate compared to the failures**  **-Post decompression**  **corrective procedures**  **-Disease severity**  **-Exophthalmometry**  **-Disease activity**  **-Adverse events**  **-QoL** | -By means of composite scores or ordinal score  -For pre-existing conditions like motility and eyelid alterations  -The NOSPECS score or the total eye score  -The clinical activity score | 1 to 6 months following surgery |
|  | Jellema et al  2015 | Proposal of success criteria for strabismus surgery  in patients with Graves’ orbitopathy based on a  systematic literature review | **-The field of BSV**  **-Quality of life**  **-GO activity** | The Goldman perimeter with the score system of Sullivan after one or two surgeries including the improvement per surgery.  -Questionnaire as developed and implemented by (Terwee et al. 1998) comprising both the visual function and appearance questions, after one or two surgeries including the change per surgery. (The GO-QoL)  -BSV, CAS score, Hertel measurement, ductions, eye position |  |
|  | Mou et al  2015 | Common Immunosuppressive Monotherapy for Graves’ Ophthalmopathy: A Meta-Analysis | -**Response rate**  **-Clinical activity score (CAS)**  **-Mean difference in proptosis**  -**Adverse events** | -Decrease in proptosis and an eyelid retraction of 2 mm or greater, an improvement in the grade of orbital soft tissue swelling, the disappearance of diplopia in the primary gaze, and/or the improvement of eye movement and visual acuity). |  |
|  | Salvi et al  2015 | Efficacy of B-Cell Targeted Therapy With Rituximab in Patients With Active Moderate to Severe Graves’ Orbitopathy: A Randomized Controlled Study | **-The clinical activity score**  **- Proptosis**  **- Lids**  **-Diplopia**  **-Eye muscle motility**  **-Quality of life score** | - The Gorman score for diplopia of 1 class or greater,  -Eye muscle ductions of 8 degrees or greater by the Foerster-Goldman perimeter, and a total motility score (TMS)  -validated disease-specific GO QoL questionnaire | At week 24 |
|  | Savino et al  2015 | Intraorbital injection of rituximab versus high dose of systemic glucocorticoids in the treatment of thyroid-associated orbitopathy | **-Disease activity**  **-Disease severity**  **-Diplopia** | -CAS  -NOSPECS  -Gorman score | 20 months |
|  | Stan et al  2015 | Randomized Controlled Trial of Rituximab in Patients With Graves’ Orbitopathy | **-Clinical activity score (CAS)**  **-Success and failure rates**  **-Proptosis**  **-Lid fissure width**  **-Diplopia score**  **-Lagophthalmos**  **-Disease severity**  **-Orbital fat/ muscle volume**  **-Quality-of-life (QOL)** | - The Gorman scale  -The Physical and Mental Component Summary scores of the Medical Outcomes Study 12-Item Short Form Health Survey (SF-12) | 24 weeks  Change between baseline and 24 or 52 weeks |
|  | Watanabe et al 2015 | Radioiodine-Associated Exacerbation of Graves’  Orbitopathy in the Japanese Population: Randomized  Prospective Study | **-Outcomes of GO** | -(1) change in diplopia of at least 1 grade; (2) change of visual acuity caused by optic neuropathy; (3) change in CAS (increase of 2 points); (4) change in MRI findings by at least 1 criterion (exophthalmos measurement change of 2 mm, enlargement of total area of the EOMs of 20%, or T2SIR of 1); and (5) ophthalmic treatment. | 1 year after RAI therapy |
|  | Taı¨eb et al  2016 | Quality of life, clinical outcomes and safety of early prophylactic levothyroxine administration  in patients with Graves’ hyperthyroidism undergoing radioiodine therapy: a randomized controlled study | **-QoL score**  **-Depression and fatigue**  **-Anxiety**  **-GO** | -The mental composite score (MCS) of the Short Form 36 (SF-36)  -The self-administered Beck Depression Inventory (BDI) and the Modified  Fatigue Impact Scale (MFIS)  -The Spielberger’s State-Trait Anxiety Inventory (STAI)  - Examination of severity using NOPECS classification and assessment of activity using the CAS | At 6 months post-RAI |
|  | Chundury et al 2016 | Orbital Radiation Therapy in Thyroid Eye Disease | **-Motility**  **-Proptosis**  **-Quality of life**  **-Compressive optic neuropathy** |  |  |

| *Sub-condition* | *Study ID* | *Title* | *Outcome measure domain* | *Outcome measurement* | *Time of measurement* |
| --- | --- | --- | --- | --- | --- |
| Myasthenia gravis  5 | Mancuso et al 2011 | Tetracycline treatment in patients with  progressive external ophthalmoplegia | -Basal lactate, AOPP, FRAP and GSH. Patients also underwent MRC, the Newcastle Mitochondrial Diseases Adult Scale (NMDAS)  **-SF-36 scale of quality of life**  **-Quantitative measurements of eye movements**  **-**Eyelid ptosis | -SF-36 scale  -Performed and videotaped by an orthoptist who measured the nine positions of gaze directly on photographs, drawing a horizontal straight line from the internal canthus  of both eyes | During each principal control (beginning of the study , after the double-blind phase (after first 3-month), after  the open-label phase 3 months, (2 weeks washout phase in between) |
|  | Benatar et al 2012 | Design of the Efﬁcacy of Prednisone in the Treatment of Ocular Myasthenia (EPITOME) trial | **-Treatment failure**  **-Efﬁcacy and safety/tolerability outcomes** | -Deﬁned as failure to achieve sustained MMS within four months of therapy, progression to GMG, or toxicity leading to discontinuation of study drug  -Study drug discontinuation, subject withdrawal and individual adverse effects | Week 36 |
|  | Benatar et al 2012 | Medical and surgical treatment for ocular myasthenia | -Development of generalised MG  **-Improvement or resolution of symptoms due to ocular myasthenia (ptosis, diplopia)**  **-Adverse events** | -Related to steroids (DM, HTN, osteoporosis, glaucoma, peptic ulcer disease) | Within 12 months of the start of the treatment |
|  | Nair et al  2014 | Ocular myasthenia gravis: A review | **-Improvement in diplopia**  **-Progression to GMG** |  |  |
|  | Benatar et al 2016 | Efficacy of prednisone for the treatment of ocular myasthenia (EPITOME): A randomized, controlled trial | **-Treatment failure**  **-Time to sustained MMS**  **-Change in ocular Quantitative Myasthenia Gravis (QMG) score**  **- Changes in quality of life** | -Deﬁned as failure to achieve sustained MMS ( minimal manifestation status),progression to GMG, or toxicity  -(NEI-VFQ-25),(NEI-VFQ-25 10-item neuro-ophthalmological ),(MG-QOL-15) | By week 16 |

| *Sub-*  *condition* | *Study ID* | *Title* | *Outcome measure domain* | *Outcome measurement* | *Time of measurement* |
| --- | --- | --- | --- | --- | --- |
| Neurogenic disorders  6 | Curi et al  2013 | VI nerve palsy (abducens palsy) | **-Ocular deviation**  **-Incomitance**  **-Function of the LR**  **-MR contracture**  **-Duction**  **-The field of binocular vision**  **-Complications** | -Scott’s force generation test  -Electrooculography or electromyography  -The forced duction test  -Degrees |  |
|  | XJ et al  2013 | Efficacy observation on electroacupuncture in the treatment of oculomotor impairment caused by ophthalmic nerve injury | **-Clinical efficacy**  **-**Palpebral fissure size  -Pupil size  **-Oculomotor range**  **-The recovery in diplopia**  (abstract only) |  | After 3 sessions of treatment |
|  | Wang et al  2015 | Effect of mecobalamin treatment on the recovery of patients with posterior communicating artery aneurysm inducing oculomotor nerve palsy after operation | **-Recovery of oculomotor nerve palsy** | -No diplopia appears when gazing whichever direction or photophobia; no ptosis: normal upward, inward and downward eye movement range, and partial or complete recovery of pupil response | A year |
|  | Engel et al  2015 | Treatment and diagnosis of congenital fourth nerve  palsies: an update | **-Hypertropia**  **-**Unilateral atrophy of the superior oblique muscle with absent trochlear nerve  **-Objectively measured head Tilt**  **-**Facial asymmetry  **-Complications** | -In PD, using Parks–Bielschowsky three-step test,(suggest change to a two-step test  increases the sensitivity)  -High-definition MRI scans  -Using a goniometer both preoperatively and  postoperatively, in degrees  -Incidence of secondary Brown syndrome |  |
|  | Singh et al  2016 | Surgical management of third nerve palsy | **-Primary position alignment**  **-**To create, centre and enlarge **the field of**  **binocular single vision**  **-Improving motility in certain**  **Cases**  **-Alleviation of abnormal head posture**  **-Elimination of diplopia** |  |  |
|  | Bi et al  2016 | Acupuncture for the Treatment of Oculomotor Paralysis: A Pilot Randomised Controlled Trial | **-Diplopia**  **-**The palpebral fissure size  **-Response rate**  **-Adverse events** | -The cervical range of motion (CROM) score  -Digital photography and digital image analysis  -Nonresponse was as follows: no improvement in the symptoms of ophthalmoplegia. | Four weeks |

| *Sub-*  *condition* |  | *Title* | ***Outcome measure domain*** | *Outcome measurement* | *Time of measurement* |
| --- | --- | --- | --- | --- | --- |
| Nystagmus  8 | Ehrt et al  2012 | Infantile and acquired nystagmus in childhood | **-Improve visual acuity**  **-Reduce the amplitude of socially embarrassing nystagmus**  **-Improvement in disabling oscillopsia and vertigo in adults**  **-Improvement in head turn in children**  **-Side effects** | -Of Pharmacological treatment (e.g. nausea, vertigo, headaches, fatigue) |  |
|  | Ehrhardt et al 2012 | Medical treatment of acquired nystagmus | **-Alleviating symptoms** (blur or oscillopsia)  **-Decrease the slow phase of UBN**  **-Improve vertical smooth pursuit**  **-Eyes are directed to point of minimum nystagmus (null point)**  **-Visual stabilization and foveation** |  |  |
|  | Claassen et al 2013 | A randomised double-blind, cross-over trial of 4-aminopyridine for downbeat nystagmus—effects on slowphase eye velocity, postural stability, locomotion and symptoms | -**Slow-phase velocity (SPV)**  **-**Stance  -Locomotion  **-Visual acuity (VA) (**Near VA)  **-Patient satisfaction**  **-Side effects** | -Recording of eye movements (3-D video oculography)  -Snellen chart with block letters at a distance  of 6 m; near VA was measured with the chart at a distance of 30 cm  - Questionnaires (adapted for this study) with Likert-scaled items to describe their satisfaction | All measurements  were done before the first drug administration (=baseline),  60 min after the first drug administration (=5 mg 4-AP  or placebo; post1) and 60 min after the last drug administration  (=10 mg 4-AP or placebo; post2) |
|  | Theodorou et al 2014  (protocol) | Non-surgical interventions for nystagmus developing in the first year of life (infantile nystagmus) | **-Binocular BCVA**  **-Estimated VA**  **-Eye movement recordings**  **-Head posture**  **-Visual recognition times**  **-Patient satisfaction and functional measurements**  **-Adverse events** | -(log MAR or Snellen)  -VEP /or eye movement recordings  -Waveform measurement  -In degrees  -e.g. (VFQ-25) questionnaires | 4-6/12 from onset of intervention |
|  | Greven et al  2014 | Four-muscle tenotomy surgery for nystagmus | **-Broadening of the null region**  **-Improved visual acuity**  **-Improved nystagmus waveforms** | -Eye movement recordings  - Eye movement recordings |  |
|  | Strupp et al  2014 | Central ocular motor disorders, including gaze palsy  and nystagmus | **-** **Head/body posture:** Tilt or turn of head/body/Position of eyelids: Ptosis  **-** **Eye position/motility:** Misalignment in primary position, spontaneous or fixation nystagmus /Horizontal or vertical misalignment  **-** Determination of **range of motility, gaze-evoked nystagmus (GEN), end-position nystagmus**  **-** **Gaze-holding function**: GEN: horizontal and vertical, rebound nystagmus  **-Slow smooth pursuit movements:** Horizontal and vertical/Smooth or saccadic  **-** **Saccades:** Horizontal and vertical when looking around or at targets /Latency, velocity, accuracy**,** conjugacy /hypo /hypermetric /saccadic intrusions/saccadic oscillations in the form of square-wave jerks/ocular flutter  **-** **Optokinetic nystagmus (OKN):Inducible,** direction, phase (reversal or monocularly  diagonal)  **-Peripheral vestibular function**  **-Fixation suppression of the VOR**  **-Peripheral vestibular spontaneous nystagmus versus central fixation nystagmus**  **-Head-shaking nystagmus**  **-Vergence test and convergence reaction** | -Inspection  -One-eye cover test/one-eye cover/uncover test/alternating cover test  -Cover/uncover test: Examination of eyes in eight positions (binocular and monocular)  -The patient is asked to track visually an object moving slowly in horizontal and vertical directions (10–20 /s) while keeping the head stationary  -Spontaneous saccades/patient is then asked to glance back and forth between two horizontal and two vertical targets. The velocity, accuracy and the conjugacy of the saccades should be noted*/ Video oculography*  -Horizontal and vertical with OKN drum or tape  -Turning the head and fixation of a target moving at same speed  -Examination with Frenzel’s glasses (magnifying lenses (+16 dioptres) with light inside, on the one hand, prevent visual fixation) Straight-ahead gaze, to the right, to the left, downward and upward  -Head-shaking test  -by moving a target from a distance of about 50 cm toward the patient’s eyes or the patient looks back and forth between a distant and a near target |  |
|  | Jayaramachandran et al 2014 | A Randomized Controlled Trial Comparing Soft Contact Lens and Rigid Gas-Permeable Lens Wearing in Infantile Nystagmus | **-Mean intensity of nystagmus at the null region viewing at 1.2 m**  **-Nystagmus foveation**  **-Best-corrected visual acuity at 4 m and 0.4 m**  **-Gaze-dependent visual acuity (GDVA)**  **-Reading performance at 0.4 m** | -Eye Movement Recordings. Eye movements were recorded on all visits using an infrared video pupil tracker with head movement  compensation (EyeLink eye tracker, SensoMotoric Instruments GmbH, Berlin, Germany) in 2 dimensions (horizontal and vertical)  -The amplitude, frequency, and intensity of nystagmus and the expanded Nystagmus Acuity (NAFX). The NAFX is an objective measure of foveation quality that can predict potential BCVA in patients without afferent visual deficits.  -Logmar  -The Radner reading chart | At 2- to 3-week intervals after each phase began |
|  | Hertle et al  2015 | Topical brinzolamide (Azopt) versus placebo in the treatment of infantile nystagmus syndrome (INS) | **-The nystagmus acuity function (NAFX)**  **-Valid measurement of visual acuity was performed with refraction in place binocularly and monocularly** | -A digital video system (EyeLink II, SR Research, Mississauga, Ontario, Canada) for the eye movement recordings.  -The standard ETDRS chart and testing method in their primary position null zone, conversion to LogMAR units was done | 2–4 weeks  after drug and placebo |

| *Sub-*  *condition* | *Study ID* | *Title* | ***Outcome measure domain*** | *Outcome measurement* | *Time of measurement* |
| --- | --- | --- | --- | --- | --- |
| Pattern deviation  5 | Akar et al  2012 | Graded anterior transposition of the inferior oblique  muscle for V-pattern strabismus | **-Changes in inferior oblique Overaction**  **-V patterns**  **-Subjective extorsion**  **-Objective extorsion**  **-Suppression**  **-Simultaneous perception fusion**  **-Anomalous retinal correspondence** |  | Postoperatively at 1, 3, 6,  12, 24, and 36 months. |
|  | Awadein et al  2013 | Lateral rectus recession with/without transposition in V-pattern exotropia without inferior oblique overaction | **-Ductions and versions in all cardinal directions of gaze**  **-Pattern strabismus**  **-Stereoacuity**  **-Degree of fundus torsion** | -Overaction was measured on 4-point scale ranging from +1 to +4  -(*Prism and alternate cover tests*), the difference between the angles of horizontal misalignment in up gaze and down gaze before surgery and at the last follow-up period  -*TNO stereo test*  -*Indirect ophthalmoscopy*, was graded on a scale of 0 to +4 as described by Guyton | A minimum follow-up of 6 months |
|  | Ghasia et al  2013 | Pattern Strabismus: Where Does the Brain’s Role End and the Muscle’s Begin? | **-Collapse the pattern**  **-Increased objective torsion**  **-Postoperative drift** |  |  |
|  | Ranka et al  2014 | Bilateral posterior tenectomy of the superior oblique muscle for the treatment of A-pattern strabismus | **-Pattern deviations**  **-Superior oblique function** | -*Prism and alternate cover* testing  was performed at both near and distance and at 30 up- and downgaze in cooperative patients  -In less cooperative patients, the  *Krimsky* method was used to determine the up- and downgaze  measurements | Preoperative assessments, 2 weeks following surgery, and at the most recent follow-up visit |
|  | Li et al  2016 | Effects of Bilateral Superior Oblique “Hang-Back” Recession in Treatment of  A-pattern Strabismus with Superior Oblique Overaction | **-Horizontal deviation** at distance, with the refractive error fully corrected and with the eyes in positions of approximately 25° upgaze and 25° downgaze  **-SOOA**  **-Torsion** | -On **a scale of +1 to +4** according to  hypotropia of the adducted eye in the tertiary position  -The **objective torsion** was measured as the angle between the line passing thro  ugh the macula and the centre of optic nerve and the horizontal line  The **corrected objective torsion** was the difference between preoperative and postoperative  torsion through the macula | A final examination was performed 6 to 9 months after surgery |

| *Sub-*  *condition* | *Study ID* | *Title* | *Outcome measure domain* | *Outcome measurement* | *Time of measurement* |
| --- | --- | --- | --- | --- | --- |
| Central causes  7 | Pollock et al  2011 | Interventions for disorders of eye movement in patients with stroke | **-Functional ability in activities of daily living**  **-Functional ability in extended activities of daily living**  **-Eye movement**  **-Balance**  **-Falls**  **-Depression and anxiety**  **-Discharge destination or residence after stroke**  **-QoL**  **-Adverse events** | -Barthel Activities of Daily Living Index etc...  Nottingham Extended Activities of Daily Living scale etc...  -Orthoptic tests  -Berg Balance Scale etc.….  -no. of reported falls, Falls Efficacy Scale  -Hospital Anxiety and Depression Scale, etc.…  -EQ5D, etc.… | Ideally 6 months |
|  | Rowe et al  2014 | Interventions for eye movement disorders due to acquired brain injury | **-Improvement in ocular motility/**  **extent of eye movement range**  **-Achievement of BSV**  **-Patient-reported symptoms**  **-Improvement in functional ability**  **-QoL**  **-Adverse events** | -Orthoptic assessments of the angle of deviation  -Cover test , motor fusional vergences /stereoacuity  -Patient record note/ questionnaires  -Validated measures |  |
|  | Boxer et al  2014 | Davunetide in patients with progressive supranuclear palsy: a randomised, double-blind, placebo-controlled phase 2/3 trial | **-PSPRS**  **-Clinical Global Impression of Change (CGIC)**  **-Brain ventricular volume** | -Six categories: daily activities, behaviour, bulbar, *ocular motor*, limb motor, and gait or midline  -MRI scans | 52 weeks |
|  | Thiagarajana et al 2014 | Oculomotor neurorehabilitation for reading in mild traumatic brain injury (mTBI):  An integrative approach | **-Near point of convergence (NPC)**  **-Near point of accommodation (NPA)**  **-Reading eye movements**  **-A subjective correlate of visual attention**  **-Symptoms related to near-work** | -Using standardized clinical procedures  -The Visagraph objective eye movement recording system  -The Visual Search and Attention Test (VSAT)  - The Convergence Insufficiency Symptom Survey (CISS) | Week 1, 8 and 15 |
|  | Strupp et al  2014 | Central ocular motor disorders, including gaze palsy  and nystagmus | **-** **Head/body posture:** Tilt or turn of head/body/Position of eyelids: Ptosis  **-** **Eye position/motility:** Misalignment in primary position, spontaneous or fixation nystagmus /Horizontal or vertical misalignment  **-** Determination of **range of motility, gaze-evoked nystagmus (GEN), end-position nystagmus**  **-** **Gaze-holding function**: GEN: horizontal and vertical, rebound nystagmus  **-Slow smooth pursuit movements:** Horizontal and vertical/Smooth or saccadic  **-** **Saccades:** Horizontal and vertical when looking around or at targets /Latency, velocity, accuracy**,** conjugacy /hypo /hypermetric /saccadic intrusions/saccadic oscillations in the form of square-wave jerks/ocular flutter  **-** **Optokinetic nystagmus (OKN):Inducible,** direction, phase (reversal or monocularly  diagonal)  **-Peripheral vestibular function**  **-Fixation suppression of the VOR**  **-Peripheral vestibular spontaneous nystagmus versus central fixation nystagmus**  **-Head-shaking nystagmus**  **-Vergence test and convergence reaction** | -Inspection  -One-eye cover test/one-eye cover/uncover test/alternating cover test  -Cover/uncover test: Examination of eyes in eight positions (binocular and monocular)  -The patient is asked to track visually an object moving slowly in horizontal and vertical directions (10–20 /s) while keeping the head stationary  -Spontaneous saccades/patient is then asked to glance back and forth between two horizontal and two vertical targets. The velocity, accuracy and the conjugacy of the saccades should be noted*/ Video oculography*  -Horizontal and vertical with OKN drum or tape  -Turning the head and fixation of a target moving at same speed  -Examination with Frenzel’s glasses (magnifying lenses (+16 dioptres) with light inside, on the one hand, prevent visual fixatioN) Straight-ahead gaze, to the right, to the left, downward and upward  -Head-shaking test  -by moving a target from a distance of about 50 cm toward the patient’s eyes or the patient looks back and forth between a distant and a near target |  |
|  | Wu et al  2015 | Wall-eyed bilateral internuclear ophthalmoplegia: review of pathogenesis, diagnosis, prognosis and management | **-Elimination of diplopia**  **-Deviations**  **-Restoration of stereopsis**  **-Reduction of abnormal**  **head posture**  **-Ability to converge**  **-Binocular single vision**  **-Motor fusion**  **-Near points of convergence** | -Arc seconds  -Worth Four Dot testing  -cm |  |
|  | Leclair-Visonneau et al 2016 | Randomized placebo-controlled trial of sodium valproate in progressive supranuclear palsy | **-Disease clinical progression**  **-Effects of VPA on cognitive and behavioural status**  **-Tolerability of the treatment**  **-Compliance** | -The PSPRS score (including ocular motor) | At 12 and 24 months |
