# Supplementary material for: Development of a core outcome set for amblyopia, strabismus and ocular motility disorders: a review to identify outcome measures
Source: BMC Ophthalmol. 2019 Feb 8;19:47. doi: 10.1186/s12886-019-1055-8 (PMC6368710; doi:10.1186/s12886-019-1055-8)
Supplement: Supplementary file 6 — Tables S4.1-4.7. Outcome measures per sub- condition of ocular motility disorder, Reported outcome measures arranged alongside OMD sub-conditions, number of studies reporting outcome measures, outcome measurements and references. (DOCX 194 kb) [file 12886_2019_1055_MOESM6_ESM.docx]

Supp. Table 4.1

| **Ocular motility disorder** | **No. of Studies reporting outcome measure** | **Outcome measure** | **Outcome measurement** | **References** |
| --- | --- | --- | --- | --- |
| **Accommodation & convergence disorders**  (7 Studies) | 5 | Near point of convergence (NPC) | Using penlight, ruler, accommodative target/ spectacle plane, bridge of nose, corneal plane, centre of rotation of eyes  (in inches or cm) | [117, 124, 125, 128, 147] |
|  | 6 | Patient symptoms | (CISS) Version-15 | [117, 123-125, 128, 147] |
|  | 4 | Positive fusional vergence at near |  | [117, 124, 125, 147] |
|  | 4 | Amplitude of accommodation | Push-up method using a moveable target of 20/30 letters on the Astron Accommodative Rule | [118, 128, 147] |
|  | 2 | Phoria | Δ (prism dioptre) | [128, 147] |
|  | 2 | Accommodative facility/BAF/MAF | Measured in cycles per minute (cpm) | [118, 147] |
|  | 1 | MEM dynamic retinoscopy |  | [147] |
|  | 1 | Fusional convergence and Fusional recovery |  | [128] |
|  | 1 | Visual Acuity |  | [147] |
|  | 1 | Refractive error |  | [147] |
|  | 1 | QoL |  | [125] |

Supp. Table 4.2

| **Ocular motility disorder** | **No. of Studies reported outcome measure** | **Outcome measure** | **Outcome measurement** | **References** |
| --- | --- | --- | --- | --- |
| **Ocular mechanical restriction**  (6 Studies) | 4 | **Resolution of diplopia** | Clinical symptoms  A field diplopia test | [105, 119, 141, 146] |
|  | 4 | **Motility assessment** | Forced duction test  Three-step test  Cover/uncover tests  Monocular (ductions) and binocular (versions)  A and V patterns  In degrees from PP | [119, 122, 129, 141] |
|  | 2 | Enophthalmos in post-traumatic orbital reconstruction | Exophthalmometer in millimetre (mm) | [141, 146] |
|  | 2 | Assessment for fractures and soft-tissue herniation (inferior rectus muscle, fat, or connective tissue) |  | [119, 122] |
|  | 1 | Orbital volume |  | [122] |
|  | 1 | Globe position |  | [122] |
|  | 1 | **Aesthetic outcome** |  | [141] |
|  | 1 | **Functional outcome** |  | [141] |
|  | 1 | **Compensatory head posture** |  | [129] |
|  | 1 | Clinical assessment | Widening of the palpebral fissure on adduction | [129] |
|  | 1 | **Visual acuity** |  | [119] |
|  | 1 | Bony contour & alignment |  | [122] |
|  | 1 | Histologic examination of cut tissue |  | [129] |
|  | 1 | **Procedure-related complications** |  | [146] |

Supp. Table 4.3

| **Ocular Motility disorder** | **No. of Studies reporting outcome measure** | **Outcome measure** | **Outcome measurement** | **References** |
| --- | --- | --- | --- | --- |
| **Myogenic disorders**  **(Thyroid eye disease, and other orbital inflammatory disorders)**  (25 Studies) | 18 | Disease activity/severity score/changes in GO | CAS  NOSPECS TAO  (TES) total eye score  Modified Werner grading scale  VISA  EUGOGO | [80-82, 88, 90, 93-97, 101, 102, 106, 107, 109-111, 114] |
|  | 14 | Change in **extraocular motility**/ Restriction of eye movements | Degrees  Subjective diplopia score (Gorman scale)  Subjective diplopia score within GO-QOL questionnaire | [80, 88, 90, 94-96, 101, 106, 107, 109, 110, 113, 114, 134] |
|  | 13 | Improvement in **quality of life score**/participant- and physician-reported global health assessment | Validated questionnaires  (SF-12)  Visual analogue scale  Sickness Index Profile (SIP)  TEDQOL  ASQ-20  (MCS) of the Short Form 36 (SF-36),and thyroid-specific  patient-reported outcome (ThyPRO) dimension scores | [80, 82, 89, 93-95, 102, 105, 107-109, 113, 114] |
|  | 8 | **Visual acuity** |  | [94-96, 99, 106, 107, 110, 134] |
|  | 7 | **Diplopia**/no diplopia in primary position and downgaze with prisms or without prisms |  | [99, 106-110, 112] |
|  | 6 | **Adverse effects** |  | [80, 82, 97, 107-109] |
|  | 5 | Number of post treatment corrective surgery including strabismus surgery |  | [81, 82, 89, 96, 107] |
|  | 4 | Proptosis/exophthalmos/  Exophthalmometry |  | [81, 106, 107, 110] |
|  | 2 | **Ocular alignment testing** | Cover tests  APCT 33 cm and 6 m  PCT 20 feet and14 inches  Krimsky test with far fixation  Synoptophore  Hess  Lees screen  calculation of duction as described by Feldon  Ductions with Kerstenbaumbrille  Maddox rod to test overcorrection >20 degrees | [108, 134] |
|  | 2 | **Binocular field of single vision** | Goldmann perimeter with the core system of Sullivan  the Harmswand  (CROM) device | [107, 108] |
|  | 2 | Lids/lid fissure width |  | [93, 114] |
|  | 2 | response rate |  | [99, 106] |
|  | 1 | **Resolution of TED symptomatology** |  | [107] |
|  | 1 | **Abnormal Head Posture** |  | [108] |
|  | 1 | **Presence of stereopsis** | Worth four dot  Titmus | [108] |
|  | 1 | Prism added |  | [108] |
|  | 1 | **Being able to drive after strabismus surgery** |  | [108] |
|  | 1 | **Appearance of the eye** |  | [107] |
|  | 1 | Orbital volume |  | [110] |
|  | 1 | Intraocular pressure |  | [110] |
|  | 1 | Corticosteroid dose reduction by at least 50% |  | [94] |
|  | 1 | reduction in pain |  | [94] |

Supp. Table 4.3

| **Ocular motility disorder** | **No. of Studies reporting outcome measure** | **Outcome measure** | **Outcome measurement** | **References** |
| --- | --- | --- | --- | --- |
| **Myogenic disorders**  **(Ocular myasthenia & external ophthalmoplegia)**  (5 Studies) | 3 | Development of generalised MG |  | [127, 133, 142] |
|  | 2 | **Improvement in diplopia** |  | [127, 133] |
|  | 2 | MMS ( minimal manifestation status) |  | [142, 143] |
|  | 2 | Change in ocular Quantitative Myasthenia Gravis (QMG) score |  | [142, 143] |
|  | 2 | **QoL** | The 25-item National Eye Institute Visual Function Questionnaire (NEI-VFQ-25)  The 10-item neuro-ophthalmological supplement to the NEI-VFQ25  The 15-item Myasthenia Gravis Quality-of Life Scale (MG-QOL-15) | [142, 143] |
|  | 1 | **Improvement in ocular symptoms** |  | [127] |
|  | 1 | Quantitative **measurements of eye movements** | Videotaped, measured the nine positions of gaze directly on photographs | [102] |
|  | 1 | Serious **adverse events** |  | [127] |

Supp. Table 4.4

| **Ocular motility disorder** | **No. of Studies reporting outcome measure** | **Outcome measure** | **Outcome measurement** | **Reference** |
| --- | --- | --- | --- | --- |
| **Neurogenic disorders**  (6 Studies) | 4 | Recovery in diplopia |  | [89, 93, 134, 144] |
|  | 4 | Eye movement/oculomotor range/ amount of duction/ incomitance |  | [89, 130, 134, 144] |
|  | 2 | Deviation | In prism dioptre | [130, 131] |
|  | 2 | Head tilt or abnormal head position | A goniometer, in degrees | [131, 134] |
|  | 2 | Increasing the field of  binocular vision |  | [130, 134] |
|  | 2 | Adverse events |  | [93, 130] |
|  | 1 | (CROM) score |  | [93] |
|  | 1 | Alignment in primary position | 3 or 2 step test (for 4th n palsy) | [134] |
|  | 1 | Assessment of MR contracture (for 6th nerve palsy) | The forced duction test | [130] |
|  | 1 | Clinical efficacy |  | [144] |
|  | 1 | Palpebral fissure size |  | [144] |
|  | 1 | Pupil size |  | [144] |
|  | 1 | Facial asymmetry (for 4th n palsy) |  | [131] |
|  | 1 | Assessment for muscle atrophy or absent nerve | high-definition MRI | [131] |
|  | 1 | Function of the LR (for 6th nerve palsy) | Scott’s force generation test Electrooculography or electromyography | [130] |

Supp. Table 4.5

| **Ocular motility disorder** | **No. of Studies reporting outcome measure** | **Outcome measure** | **Outcome measurement** | **References** |
| --- | --- | --- | --- | --- |
| **Nystagmus**  (8 Studies ) | 6 | **VA/Binocular BCVA/**  **Estimated VA** | LogMAR or Snellen  Pattern reversal VEP | [85-87, 120, 126, 145] |
|  | 4 | **Eye movement recordings** | Video  Infrared limbal tracker  Infrared video pupil tracker  Electro-oculogram  3-D video-oculograph  Waveform measurement including foveation  Expanded nystagmus acuity function (NAFX) | [85-87, 126] |
|  | 2 | Improvement in oscillopsia and vertigo in adults |  | [120, 137] |
|  | 2 | **Improve head posture** | Measured in degrees | [120, 126] |
|  | 2 | **Patient satisfaction and functional measurements** | VFQ-25 | [87, 126] |
|  | 2 | Broadening of the null region/visual stabilization and foveation/eyes are directed to null point | Eye movement recordings | [137, 145] |
|  | 1 | Reduce the amplitude of nystagmus |  | [120] |
|  | 1 | Decrease the slow phase of UBN and improve vertical smooth pursuit |  | [137] |
|  | 1 | Improved nystagmus waveforms | Eye movement recordings | [145] |
|  | 1 | Visual recognition times |  | [126] |
|  | 1 | Mean intensity of nystagmus at 1.2 m/at 0.4 m |  | [86] |
|  | 1 | **Determination of range of motility**, gaze-evoked nystagmus (GEN), end-position nystagmus |  | [132] |
|  | 1 | Gaze-holding function: GEN: horizontal and vertical, rebound nystagmus |  | [132] |
|  | 1 | Optokinetic nystagmus (OKN): Inducible, direction, phase (reversal or monocularly diagonal) | Horizontal and vertical with OKN drum or tape | [132] |
|  | 1 | Fixation suppression of the VOR |  | [132] |
|  | 1 | Peripheral vestibular spontaneous nystagmus versus central fixation nystagmus | Frenzel’s glasses | [132] |
|  | 1 | Head-shaking nystagmus | Head-shaking test | [132] |
|  | 1 | **Adverse events** |  | [126] |

Supp. Table 4.6

| **Ocular motility disorder** | **No. of Studies reporting outcome measure** | **Outcome measure** | **Outcome measurement** | **Reference** |
| --- | --- | --- | --- | --- |
| **Patterns deviation**  (5 Studies) | 4 | **Collapse the pattern** | Prism dioptres (Δ)  Prism and alternate cover testing in up and down gaze  Krimsky method in less cooperative patients | [136, 138-140] |
|  | 4 | **Objective torsion** | Indirect ophthalmoscopy | [121, 136, 139, 140] |
|  | 2 | **horizontal deviation** at distance, with the refractive  error fully corrected and with the eyes in positions of  approximately 25° upgaze and 25° downgaze |  | [139, 140] |
|  | 2 | **Superior oblique function** | A scale of +1 to +4 | [138, 139] |
|  | 1 | **Inferior oblique overaction** | A scale of -4 underaction to+4 overaction, with 0 being normal | [121] |
|  | 1 | **Subjective extorsion** | Double Maddox rod test | [121] |
|  | 1 | **Stereoacuity**, **anomalous retinal correspondence, simultaneous perception, suppression** | TNO stereo test, major amblyoscope, Worth 4-dot tests | [121] |
|  | 1 | **Postoperative drift** |  | [136] |

Supp. Table 4.7

| **Ocular motility disorder** | **No. of Studies reporting outcome measure** | **Outcome measure** | **Outcome measurement** | **Reference** |
| --- | --- | --- | --- | --- |
| **Central causes**  (7 Studies) | 3 | **Range of motility** | Cover/uncover test  Gradation of movements | [80, 81, 132] |
|  | 2 | **Deviations** | One-eye cover test/one-eye cover/uncover test/alternating cover test | [106, 132] |
|  | 2 | **Reduction of abnormal head posture** | Inspection | [106, 132] |
|  | 2 | PSPRS | A scoring system used to assess 28 signs and symptoms of PSP in six categories: daily activities, behaviour, bulbar, **ocular motor,** limb motor, and gait or midline | [88, 94] |
|  | 1 | **Resolution of diplopia in primary gaze** |  | [106] |
|  | 1 | **Near point of convergence** | cm | [106] |
|  | 1 | **Restoration of stereopsis** | Arc seconds | [106] |
|  | 1 | **Motor fusion** |  | [106] |
|  | 1 | **Nystagmus: GEN, UBN,DBN, fixation type** | Video-oculography  Head-impulse test | [132] |
|  | 1 | **Pursuits and saccades** | The optokinetic drum  Video-oculography | [132] |
|  | 1 | **Patient-reported symptoms** | patient record notes or questionnaires | [80] |
|  | 1 | **QoL** |  | [80] |
|  | 1 | **Achievement of BSV** | cover test , motor fusional vergences and stereoacuity | [80] |
|  | 1 | **Near point of accommodation(NPA)** | Push-up method | [100] |
|  | 1 | **Reading eye movements** | Visagraph objective eye movement | [100] |
|  | 1 | **Binocular horizontal versional eye movements** | Arrington eye movement recording system | [100] |
|  | 1 | **Saccade ratio** | Calculated using a simulated reading single line (SRSL) (±5◦ horizontal range) and a simulated reading multiple line (SRML) | [100] |
